# Relay learning: a physically secure framework for clinical multi-site deep learning

**DOI:** 10.1038/s41746-023-00934-4

**Published:** 2023-11-04

**Authors:** Zi-Hao Bo, Yuchen Guo, Jinhao Lyu, Hengrui Liang, Jianxing He, Shijie Deng, Feng Xu, Xin Lou, Qionghai Dai

**Affiliations:** 1 School of Software, Tsinghua University, Beijing, China; 2 BNRist, Tsinghua University, Beijing, China; 3 Department of Radiology, Chinese PLA General Hospital / Chinese PLA Medical School, Beijing, China; 4 Department of Thoracic Oncology and Surgery, China State Key Laboratory of Respiratory Disease & National Clinical Research Center for Respiratory Disease, The First Affiliated Hospital of Guangzhou Medical University, Guangzhou, China; 5 Department of Radiology, The 921st Hospital of Chinese PLA, Changsha, China; 6 Department of Automation, Tsinghua University, Beijing, China

**Keywords:** Experimental models of disease, Computational platforms and environments

## Abstract

Big data serves as the cornerstone for constructing real-world deep learning systems across various domains. In medicine and healthcare, a single clinical site lacks sufficient data, thus necessitating the involvement of multiple sites. Unfortunately, concerns regarding data security and privacy hinder the sharing and reuse of data across sites. Existing approaches to multi-site clinical learning heavily depend on the security of the network firewall and system implementation. To address this issue, we propose Relay Learning, a secure deep-learning framework that physically isolates clinical data from external intruders while still leveraging the benefits of multi-site big data. We demonstrate the efficacy of Relay Learning in three medical tasks of different diseases and anatomical structures, including structure segmentation of retina fundus, mediastinum tumors diagnosis, and brain midline localization. We evaluate Relay Learning by comparing its performance to alternative solutions through multi-site validation and external validation. Incorporating a total of 41,038 medical images from 21 medical hosts, including 7 external hosts, with non-uniform distributions, we observe significant performance improvements with Relay Learning across all three tasks. Specifically, it achieves an average performance increase of 44.4%, 24.2%, and 36.7% for retinal fundus segmentation, mediastinum tumor diagnosis, and brain midline localization, respectively. Remarkably, Relay Learning even outperforms central learning on external test sets. In the meanwhile, Relay Learning keeps data sovereignty locally without cross-site network connections. We anticipate that Relay Learning will revolutionize clinical multi-site collaboration and reshape the landscape of healthcare in the future.

## Introduction

Big data combined with artificial intelligence (AI) has facilitated numerous applications in the past few years, including AI-aided medicine^1,2^. Many powerful medical AI systems have been built for clinical applications^3–5^. For example, by gathering hundreds of thousands of training samples from multiple clinical sites, expert-level performance has been reported for intracranial hemorrhage^6^, fundus disease detection^7^, lung cancer screening^8^, etc.

However, besides the power and importance, big data also brings non-negligible risks and challenges. The data privacy and security issue sabotages directly collecting big data from multiple sites and thus limits the wide application of AI technologies^1^. To handle this problem, techniques like Federated Learning^9,10^, and Swarm Learning^11^ are proposed. These techniques just require the data of each site to be put online and they use the data to train models locally and share only the parameters of the models but not the data among sites. This parameter-sharing represents a significant milestone in privacy-preserving tasks. Nonetheless, such methods require the medical data to be connected and exposed to the Internet topologically during the training as consistent model training and frequent model parameter transmission are necessary. As there is a risk that the network firewall gets attacked, the data may be disclosed by the attacks. Furthermore, the training step usually takes days or weeks, which adds to the risk. Due to the data disclosing risk, some individuals and organizations strictly forbid data and information online to ensure security. This case happens frequently in medical applications, where data privacy is crucial. In general, the current AI technologies cannot fully handle the data privacy and security issues in leveraging big data.

We introduce Relay Learning, a de-connection solution that provides physical security to data sovereignty in clinical multi-site deep learning. It disconnects all the participants physically, where only the model is delivered over clinical sites one by one like in a relay race. To be specific, the model transmission is only performed once for one site after the model training, and since the training is accomplished, the medical data can be physically disconnected from the model when the transmission happens, which makes full protection of the medical data. The asynchronous model transmission also reduces cooperation costs, where sites can join and perform model training at any time as the relay order does not affect the performance of the relay learning technique.

**Fig. 1:**
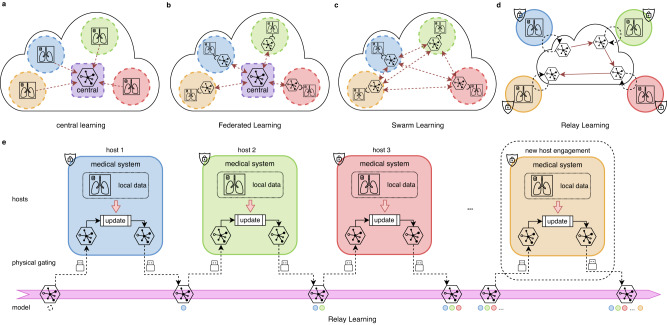
Overview of Relay Learning with other multi-site deep learning solutions. **a** In central learning, multi-site data is aggregated to a central host, resulting in complete privacy leakage. **b** Federated Learning and **c** Swarm Learning use an online distributed-computing strategy where only model parameters or gradients are transported, either in a centralized or decentralized manner. However, data servers and medical systems are still connected physically and the parameter transmission is synchronous and frequent. **d** We propose Relay Learning, a physically secure multi-site framework, which disconnects data servers from the computing topology physically. **e** In Relay Learning, the model is updated using local data inside each host sequentially to acquire cumulative ability. Hosts are physically isolated from each other. Only the model is transported through controllable hardware. New hosts can participate in Relay Learning dynamically without any interruption to the existing computation.

The framework of Relay Learning, together with other multi-site approaches, is illustrated in Fig. 1. In local learning, each institution learns the model on its own data separately, without the benefit of large-scale multi-site data. Central learning introduces a central node that collects data directly from all the participants, which violates privacy regulations. Federated Learning requires the parameter gradients to be gathered to a central node online and frequently for training. Swarm Learning modifies Federated Learning to a decentralized version, therefore the potential security risk still exists. To address the aforementioned problems, Relay Learning disconnects the clinical site offline from the computation topology by passing only the model asynchronously, and only once after training in each site. This procedure happens sequentially at each host in Relay Learning: the deep model is updated inside, copied from the computation topology, and carried out from the host to the next. The ability of Relay Learning to maintain and chronologically update knowledge is facilitated by a generative relay system (see “Methods” section for details). The relay system learns the data distribution in each site without storing data. Then the knowledge of previous sites can be reviewed by sampling from the learned distribution, and thus the multi-site knowledge can be merged given the generative relay system. The physical gating in Fig. 1d transfers the model across sites via storage devices, or via the Internet after physically and securely disconnected from the data. This controls the clinical sites isolated and disclosed from interlopers.

We hypothesize that in real-world clinical multi-site deep learning, there is a dilemma that institutions should keep their data locally while data reuse and sharing are still needed to build powerful models in this big-data era. To address this issue with a flexible and secure multi-site deep learning framework, we propose a de-connection solution that makes full use of multi-site medical data. Relay Learning guarantees that: (1) medical institutions can be completely disconnected from the Internet and become isolated systems; (2) data sovereignty is physically controlled inside hospitals away from interlopers; (3) participants are flexible to join the computation at any time; (4) transmission cost is minimized by passing only the final model in each site; (5) cooperation cost is minimized without the requirement for the sites to be synchronous online during training; (6) the framework is decentralized and the increase in transmission cost is linear with the increase in the number of institutions; (7) the connection topology order of sites is flexible; (8) complicated clinical tasks are supported, e.g., pixel-wise lesion localization on 3D medical images, due to its generalized compatibility for advanced deep learning models. Relay Learning enables clinical deep learning models to fuse the broad knowledge offline from multi-site data without privacy leakage, which is key in this big-data era.

## Results

### Experimental settings

In this research, we evaluated Relay Learning in different multi-site clinical problems for different diseases and anatomical structures. First, we tested Relay Learning to segment important retina fundus structures on several widely used public datasets, including five hosts. In addition, we dedicatedly collected a large 3D CT dataset from eight institutions and used Relay Learning to develop a multi-site simulation system for mediastinum tumor diagnosis. Finally, we managed to deploy Relay Learning in five medical institutions and trained a deep model to perform brain midline localization from medical images. The trained model was also sent to another three sites for the external test. Since the brain midline localization task was deployed in the real world, data sovereignty was kept locally in each site, which is impossible for central learning. In each internal host of all these three tasks, data samples were subject-wisely split for training and testing. Then all the samples in external hosts were used for testing. The dataset details and preprocess, the settings of Relay Learning, and the deep models for different tasks are described in the “Methods” and Supplementary Notes sections.

We conducted a variety of evaluations to fully assess the performance of Relay Learning compared to other approaches. In each task, we first compared Relay Learning with local learning, which was trained on a single host and tested on all the test sets. Next, in the first two tasks, we compared our framework with central learning (Fig. 1a), because the objective of Relay Learning is to provide physical security, while still achieving comparable performance to directly gathering data. Federated Learning and Swarm Learning can also be considered as distributed simulations of central learning, though their variations in parameter aggregation strategies may decrease their performance compared to central learning in some situations. In the last task of localizing brain midlines, we cannot evaluate a central learning model because data is kept locally. Furthermore, we built a baseline for our framework—training the task model sequentially through hosts but without the relay system. The sequential method, which can be considered a sequential fine-tuning strategy, is widely adopted as a transfer learning routine in cross-dataset deep learning. It may forget the previous knowledge rapidly^12^, thus becoming an unpractical solution in multi-site clinical applications. At last, to evaluate the influence of the host order in the training topology, besides the main sequential order (episode1), Relay Learning and sequential learning were also conducted in a reversed order (episode2) and another random order (episode3).

### Segmenting retina fundus structures

Retina fundus image is important in ophthalmic diagnosis and useful to observe the homeostasis of the human body^13^. One important biomarker for many diseases is the biomedical structure of the retina fundus, including the optic cup(OC), optic disc(OD), and other structures^14^. Here, we evaluated Relay Learning on a public OC/OD segmentation dataset of retinal fundus images, which consists of four internal hosts (F1–F4)^15–17^ and an external test host (F5)^18^. As a medical segmentation task, we used the widely adopted Dice similarity coefficient (Dice) as our evaluation metric (higher is better), which was computed image-wisely and averaged on OC and OD. We also show 95% CI (confidence interval) of the mean evaluation score in the analysis.

**Fig. 2:**
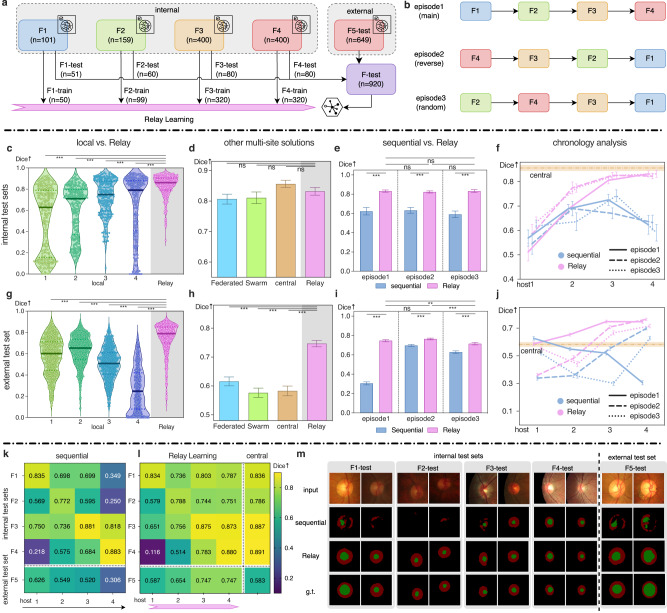
Relay Learning segments retina fundus structures. We compared Relay Learning to local learning, Federated Learning, Swarm Learning, central learning, and a sequential fine-tuning strategy. **a** Four internal institutions (F1-F4) and one external institution (F5) were incorporated, including 1709 retina fundus images. **b** Sequential and Relay Learning were evaluated in three episodes, including a forward, a backward, and a random order. If not specified, we show the result in the main episode. We show analysis on the internal and external test sets in the second row (**c**–**f**) and third row (**g**–**j**) respectively. **c**, **g** Performance violin plot of Relay Learning and four local learning models. Centerline: median; dotted lines: 1st and 3rd quartiles; dot spots: images in *F*-test. **d**, **h** Comparison of Federated Learning, Swarm Learning, central learning, and Relay Learning. **e**, **i** Comparison of sequential learning and Relay Learning in three episodes. **f**, **j** Chronology analysis of sequential and Relay Learning during the training pipeline (horizontal axis) in three episodes. **k**, **l** Dice score heatmaps for sequential and Relay Learning during the training pipeline (horizontal axis) on each test set (vertical axis). **m**, Qualitative evaluation for sequential and Relay Learning compared to the ground truth, where samples were selected randomly from each test set. Dice values are averaged on OC and OD. The mean Dice scores are shown with 95% CI bars in (**d**–**f**) and (**h**–**j**). *n*: number of fundus images; *↑*: higher is better; g.t. ground truth; ns: *P* ≥ 0.05; ^⋆^*P* < 0.05; ^⋆⋆^*P* < 0.01; ^⋆⋆⋆^*P* < 0.001. Please refer to the “Methods” section for statistical details.

Results shown in Fig. 2 demonstrate the supremacy of Relay Learning compared to local and sequential learning. Relay Learning achieved Dice values of 0.832 (0.819–0.845, 95% CI) on internal test sets and 0.747 (0.736–0.758, 95% CI) on the external test set, which were 0.211 and 0.252 higher than those of local learning (Fig. 2c, g). Compared to sequential learning, Relay Learning still maintained 0.213 and 0.198 Dice superiority on internal and external test sets averaged on three episodes. The host order did not affect the performance much (Fig. 2f, j) as shown by a further experiment of all the permutations of the host order including 24 episodes (Supplementary Fig. 1 and Supplementary Table 8), where Relay Learning still kept the performance evenly in all the episodes. We also observed that Federated Learning and Swarm Learning showed a slight decrease compared to central learning (Fig. 2d, h), except for Federated Learning on the external test set. This phenomenon may be caused by their sparser parameter-aggregation strategy compared to central learning. In Fig. 2d, Relay Learning achieved comparable performance to central learning on internal test sets (*P*-values 0.348, 0.116, and 0.362 for the three episodes respectively). Moreover, on external test sets (Fig. 2h), Relay Learning even exceeded naive central learning (0.164, 0.182, and 0.133 Dice increment on three episodes, *P*-values all < 0.001), which demonstrates its better generalization ability. This can be explained by that the relay system models the knowledge of previous sites as a distribution, which may intrinsically extend the information of the original samples and improve the generalization ability of the trained model.

The Retina Fundus dataset contains diverse data distributions across sites (see in Fig. 2m and Supplementary Fig. 2). This brings training difficulties to multi-site deep learning models— both the local learning and the sequential learning struggled in this situation (Fig. 2c–j). In Fig. 2f, j, the sequential model cannot keep improving its performance with the training on new hosts and even drop its performance greatly at the last host. This reveals that without dedicated optimization, sequential training is inadequate in multi-site settings, especially when data distribution varied much across sites. On the other hand, Relay Learning kept its performance increasing during the training on different hosts as demonstrated by the results shown in Fig. 2k, l. To be specific, in Fig. 2m, we can see sequential learning can only predict acceptable results on the last seen host F4, indicating that sequential learning forgot the knowledge learned from previous hosts. Relay Learning did not suffer from this distribution-shift problem as the generative model in our relay system learned all the previous data distribution. The robustness of Relay Learning demonstrates its potential in real-world clinical applications. Detailed results (including Dices for OC and OD seperately) and *P*-values are shown in Supplementary Tables 2, 3, 6, and 7.

### Diagnosing mediastinum tumors

**Fig. 3:**
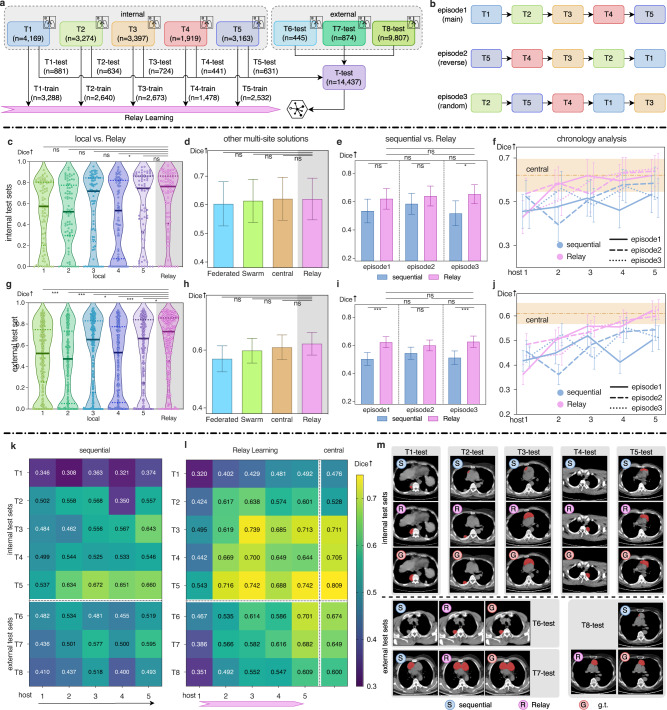
Relay Learning segments mediastinum tumors. We compared Relay Learning to local learning, Federated Learning, Swarm Learning, central learning, and a sequentially fine-tuning strategy. **a** Five internal hosts (T1–T5) and three external hosts (T6–T8) were incorporated, including 27,048 2D image slices. **b** Sequential learning and Relay Learning were evaluated in three episodes, including a forward, a backward, and a random order. If not specified, we show the result of the main episode. We show analysis on the internal and external test sets in the second row (**c**–**f**) and third row (**g**–**j**) respectively. **c**, **g** Performance violin plot of Relay Learning and five local learning models. Centerline: median; dotted lines: 1st and 3rd quartiles; dot spots: CT instances in *T*-test. **d**, **h** Comparison of Federated Learning, Swarm Learning, central learning, and Relay Learning. **e**, **i** Comparison of sequential learning and Relay Learning in three episodes. **f**, **j** Chronology analysis of sequential learning and Relay Learning during the training pipeline (horizontal axis) in three episodes. **k**, **l** Dice score heatmaps for sequential learning and Relay Learning during the training pipeline (horizontal axis) on each test set (vertical axis). **m** Qualitative evaluation for sequential learning and Relay Learning compared to the ground truth, where samples were selected randomly from each test set. The mean Dice scores are shown with 95% CI (confidence interval) bars in (**d**–**f**) and (**h**–**j**). *n*: number of 2D image slices; *↑*: higher is better; g.t. ground truth; ns: *P* ≥ 0.05; ^⋆^*P* < 0.05; ^⋆⋆^*P* < 0.01; ^⋆⋆⋆^*P* < 0.001. Please refer to the “Methods” section for statistical details.

After using a public dataset, here we collect real clinic data in an unbiased manner to mimic the real case of applying Relay Learning. The clinical task here is diagnosing mediastinum tumors. Mediastinum is the anatomic region located between the human lungs, which contains most of the primary tissues and organs in the chest except the lungs. In recent decades, the incidental encounters of mediastinum tumors increase in both clinical practice and the screening of lung cancer, with the wide usage of computed tomography (CT)^19^. The tumor states vary greatly in location, composition, and imaging characteristics^20^, which leads to the difficulty to identify tumors from CT scans. Here, we used Relay Learning to build a mediastinum tumor diagnosing system to evaluate its applicability in this challenging task of identifying lesion regions. We collected a multi-site Mediastinum Tumor dataset with CT images captured from eight medical sites (T1–T8), including five internal hosts (T1–T5) and three external hosts (T6–T8). The images in the external hosts were used for the external test (Fig. 3a). A total of 27,048 2D image slices were incorporated. We also used Dice to evaluate the performance in this use case, which was computed subject-wise. This unbiased clinical dataset is composed of all the images containing mediastinum tumors during a period of time in each hospital, thus it can directly reflect the ability of Relay Learning in real clinical usage.

The Dice result is shown in Fig. 3. Relay Learning achieved 0.638 (0.598–0.678, 95% CI) and 0.616 (0.593–0.638, 95% CI) on the internal and external test sets averaged on three episodes, which is much higher than local learning by 0.113 and 0.124 (Fig. 3c, g). Relay Learning also overtook sequential learning on internal and external test sets by 0.092 and 0.097 respectively, averaged on three episodes (Fig. 3e, i). Besides, the host order did not affect the performance of Relay Learning much as shown in Fig. 3f, j. Federated Learning and Swarm learning were still worse than central learning (Fig. 3d, h), though the gaps were small. Moreover, Relay Learning even slightly overtook central learning by 0.017 and 0.006 on the internal and external test sets respectively, averaged on three episodes. This means Relay Learning can achieve acceptable or even better performance without collecting sensitive data to a central node.

The evaluation demonstrates the robustness and generalization of Relay Learning in a multi-site clinical task. According to host chronology analysis in Fig. 3f, j, the performance of Relay Learning raised gradually when trained on more hosts and finally approached the performance of central learning, while sequential learning was quite unstable when switching to some hosts. Specifically, Relay Learning outperformed the sequential strategy on all eight test sets (Fig. 3k, l). The performance of the sequential learning dropped quickly after training on some hosts, such as T2-test (dropped 0.218) and T8-test (dropped 0.118) after training on host4 (T4-train), which may be due to the different data distribution on these hosts and the knowledge forgetting in sequential fine-tuning^21^. In addition, the performance of sequential learning dropped by 0.032 from the internal to the external test sets, while Relay Learning did not change much (slightly increased by 0.002). The result indicates that Relay Learning can utilize multi-site data more efficiently and has better generalization ability. Detailed results and *P*-values are shown in Supplementary Tables 4, 6, and 7.

### Locating brain midlines

After collecting unbiased clinical data to mimic the real case, here we do not collect data but directly use the data in their original sites to deploy Relay Learning, which is a real-world multi-site clinical experiment. Eight institutions were incorporated, including three external test sites. The deep learning model was trained to locate brain midlines from CT images. The human brain can be grossly divided into a pair of hemispheres symmetrically in size and shape by the brain midline. The brain midline shift (MLS) is an important indicator of numerous brain abnormalities, including traumatic brain injury (TBI), tumor, stroke, herniation, and other severe intracranial lesions^22^. Therefore, the localization of the brain midline and the measurement of MLS can help the diagnosis. We managed to deploy Relay Learning in five internal institutions (M1–M5) and train a deep model that can locate brain midlines. The model was tested on three external institutions (M6–M8). The incorporated multi-site Brain Midline dataset had 12,281 2D head CT images in total and was held inside each site locally, therefore no central model was trained. The brain midline is a line-shaped target, which differs from other common biomedical structures and lesions. To measure the distance between the predicted midline and the ground truth, we used Hausdorff Distance (HD) on each slice as our metric (lower is better). In this use case, we deployed Relay Learning to real-world clinical usage for identifying biological structures.

We used the Relay Learning framework to train a segmentation model to locate brain midlines on our multi-site Brain Midline dataset. The model was sequentially updated and transported across the five institutions using portable storage devices. The trained model was finally delivered to the three external sites for testing. The performance was calculated in each site with its own test data.

**Fig. 4:**
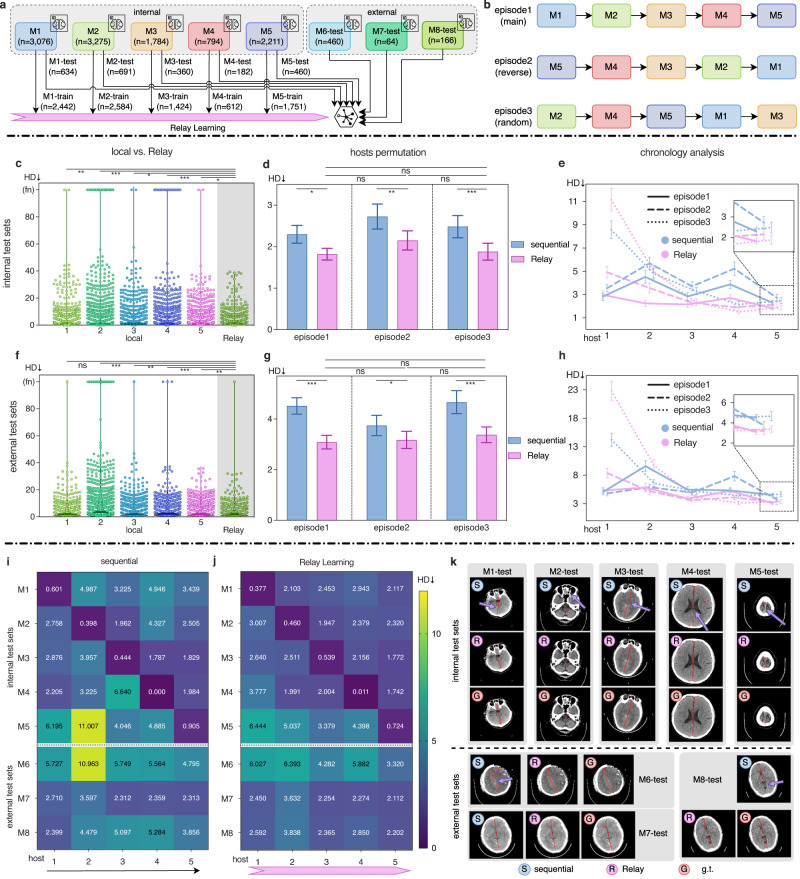
Relay Learning locates brain midlines. In this clinical deployment, data was hosted inside each site and only task models were delivered across sites, where central learning is impossible. We compared Relay Learning to local learning and a sequentially fine-tuning strategy. **a** Five internal (M1–M5) and three external institutions (M6–M8) were incroporated, including 12,281 2D image slices. **b** Sequential learning and Relay Learning were evaluated in three episodes, including a forward, a backward, and a random order. If not specified, we show the result in the main episode. We show analysis on the internal and external test sets in the second row (**c**, **d**, **e**) and third row (**f**, **g**, **h**) respectively. **c**, **f** Performance scatter plot of Relay Learning and five local learning models. dot spots: CT slices in M-test; fn: false-negative predictions where the model fails to predict any midlines. **d**, **g** Comparison of Relay Learning and sequential learning in three episodes. **e**, **h** Chronology analysis of sequential learning and Relay Learning during the training pipeline (horizontal axis) in three episodes. **i**, **j** HD score heatmaps for sequential learning and Relay Learning during the training pipeline (horizontal axis) on each test set (vertical axis). **k** Qualitative example visualization for sequential learning and Relay Learning compared to the ground truth, where samples were selected randomly from each test set. The mean HD scores are shown with 95% CI bars in **d**, **e**, **g**, **h**. *n*: number of 2D image slices; *↓*: lower is better; g.t. ground truth; ns: *P* ≥ 0.05; ^⋆^*P* < 0.05; ^⋆⋆^*P* < 0.01; ^⋆⋆⋆^*P* < 0.001. Please refer to the “Methods” section for statistical details.

In Fig. 4c, local learning failed to predict reasonable brain midlines in numerous samples (4, 28, 2, 16, and 2 failure samples for the five local models respectively on internal test sets), while the performance of Relay Learning was better with fewer failures (0 in internal and 1 in external test sets). Relay Learning achieved HD scores of 1.819 (1.678–1.960, 95% CI) and 3.090 (2.821–3.358) on internal and external test sets in the main episode, which were better than local learning by 40.2% and 41.9% respectively. Relay Learning also overtook sequential learning by a large margin (22.1% and 25.4% on internal and external test sets). As Fig. 4e and h show, Relay Learning rapidly outperformed sequential learning at the 3rd host. Detailed metrics on each test set of the five internal hosts (Fig. 4i, j) indicate that sequential learning and Relay Learning performed well on the specific test set right after being trained on that host (see in diagonal). However, sequential learning dropped quickly in the following hosts, while Relay Learning showed a smaller performance drop. Besides, Relay Learning outperformed sequential learning on all three external test sets. Qualitative results (Fig. 4k) show that the sequential model often generated irrelevant (M2, M4, and M6) and interrupted (M1, M3, M6, and M8) lines, while Relay Learning extracted similar midlines as the ground truth. This result demonstrates in actual clinical systems, Relay Learning performs consistently well. Detailed results and *P*-values are shown in Supplementary Tables 5, 6, and 7.

## Discussion

To the best of our knowledge, Relay Learning is the first physically secure multi-site deep learning framework capable of making full use of multi-site medical data without any physical connections between the data sites and the Internet. This physical security makes medical institutions, hospitals, and other organizations get controllable data sovereignty and confidentiality to fully contest malicious entities. In the meantime, it makes the modern deep learning models benefit from multi-site training and thus achieve similar performance to central learning with physical data aggregation. Specifically, the relay system learns to model the continuous data distribution locally and externally re-samples from that distribution to get virtual individuals different from any real identities in all the hosts, thus containing no personal information and protecting data privacy. Although it does not store any actual data, the relay system is significant in multi-site learning because the learned data distribution of different hosts can largely help to train the model to achieve high robustness and generalization ability. In real-world applications, data often follows diverse patterns across clinical sites due to diverse image-acquiring conditions, by learning the data distribution rather than keeping the original samples, our relay system keeps data security and privacy in a de-connection manner and still fully benefits from the multi-site data.

Relay Learning draws lessons from recent advances in deep learning. Aiming at modeling the data distribution, generative adversarial network (GAN)^23^ has shown great potential in various tasks^24–26^. Its generative modeling is domain-specific and identity-free, which inspires us to use it in clinical data sharing in a privacy-safeguarded manner^27–29^. We managed to improve current advanced GANs to a DoubleGAN-based relay system, which is suitable for pixel-wise tasks, and addressed a sharp transition problem in Relay Learning (see the “Methods” section for details). The sequential training procedure also appears in continual learning^21^. However, many well-performed continual learning methods store a partial dataset for future tasks, which directly violets the privacy requirement in clinical settings^30,31^. In addition, most of them are assessed in tasks such as image classification^32,33^, and are still immature in challenging clinical tasks, like pixel-level lesion segmentation (see Supplementary Results section and Supplementary Fig. 3 for a comparison). Relay Learning leverages a pixel-level generative relay system to completely model data distribution, enhancing its ability to handle complex segmentation tasks.

In the era of AI-aided precision medicine and medical big data, Relay Learning focuses on physically secure learning in multi-site data utilization, compared to some relevant multi-site approaches. In Federated Learning or Swarm Learning, the model parameters rather than the data are exchanged frequently during the model training. Even though this strategy does not leak the data, as the data is also accessible by the model during the training, there is a risk of data leaking by directly attacking the model through the internet or by inversion attacks which can recover personal statistics through parameter gradients^34,35^. This could damage data confidentiality and increase distrust among participants. Besides, the frequent online transmission and the synchronous training strategy increase the cost of multi-site cooperation. Relay Learning introduces a completely de-connection method, where clinical data are physically disconnected from the network. Relay Learning also fixed extra issues in these methods: Federated Learning brings a central node which may cause data monopolies; Swarm Learning implicates edge computing with the exponential increase in computation complexity when adding new nodes.

There are several limitations of this study. First, the introduction of DoubleGAN increases computational time. This is a drawback of such an asynchronous sequential method compared to synchronous Federated and Swarm Learning, though Relay Learning can ensure physical security and minimize communication costs. We managed to reduce the training time with an efficient model design. In the Relay Learning pipeline, except for the first host, the training of DoubleGAN in subsequent hosts is a finetuning procedure, which can reduce the convergence time largely. Besides, The training trajectory of Relay Learning is sequential, which brings advantages such as the linear computation increment and the flexibility for new host engagement. The evaluation result also shows that the performance of Relay Learning was relatively consistent in different host orders. However, the hosts are treated differently in theory due to the sequential procedure, which may affect fairness in some particular applications. We can partially fix this issue by ensembling models trained with multiple host orders or designing the training topology/order according to conditions and objectives in different applications.

Cross-site and international sharing of medical data are essential for modern healthcare, especially for rare diseases^36^. However, the strict privacy provision and ethics certification limit direct data transfer and remote access. Instead of data or parameter online sharing that may encompass the message of personal information, we suggest reusing and sharing depersonalized knowledge offline in AI models. We anticipate that the deployment of Relay Learning would help to encourage innovation of AI-aided solutions in medicine, respect human rights in AI systems, promote healthcare resource-sharing, improve the fairness of data governance, and revolutionize the collaboration of clinical sites, groups of hospitals, and international organizations in biomedical and healthcare research, especially in this turbulent global condition with regional disputes and other international threats.

## Methods

### Datasets

We used three multi-site datasets in the evaluation of Relay Learning, including the Retina Fundus dataset, Mediastinum Tumor dataset, and Brain Midline dataset. The samples in the Brain Midline dataset were held inside each clinical site, while samples in the other two datasets were gathered to simulate the multi-site learning.

#### Retina Fundus dataset (F1-F5)

The public Retina Fundus dataset were split into five hosts – Drishti-GS (F1)^15^, RIM-ONE-r3 (F2)^16^, REFUGE train (F3)^17^, REFUGE test (F4)^17^, and ORIGA-light (F5)^18^. Due to different imaging conditions, the data from each clinical host shows varying distributions. The five hosts contain 101, 159, 400, 400, and 649 fundus images respectively. We followed the data split strategy in previous work for the four internal sets^37^: samples in F1 and F2 were split into training and test sets as the original providers, which are 50 + 51 for F1 and 99 + 60 for F2, while samples in F3 and F4 were randomly partitioned at a ratio of 4:1, which are both 320 + 80. Samples in F5 were all used for external test. We preprocessed the fundus images similarly to a previous work^38^. The original images and the corresponding OC/OD annotations were center-cropped to the disc region uniformly at a resolution of 800 × 800. The cropped images and annotations were then resized to 64 × 64 before being fed into the deep neural network. The RGB input images were normalized uniformly to the range [−1, 1], and the annotation maps were considered as three-class segmentation masks. More details are shown in Supplementary Table 1.

#### Mediastinum tumor dataset (T1–T8)

We collected a Mediastinum tumor dataset consisting of chest CT images from eight different institutions, including The First Affiliated Hospital of Guangzhou Medical University (T1), The People’s Hospital of Gaozhou (T2), Sichuan Cancer Hospital and Institute (T3), Zhongshan People’s Hospital (T4), Affiliated Cancer Hospital and Institute of Guangzhou Medical University (T5), The First Affiliated Hospital of Xi’an Jiaotong University (T6), The Fourth Hospital of China Medical University (T7), and Shanghai Chest Hospital (T8). This dataset is a part of CAIMEN^39^. Each institution conducted a retrospective search from the local picture archiving and communication system (PACS) database for all the plain lung CT scans from Jan. 1st, 2010 to Oct. 31st, 2020 whose report includes at least one of the following terms: “mediastinal nodule", “mediastinal lesion", “mediastinal neoplasm", and “mediastinal mass". The data from different sites share the same inclusion period that reflects the natural prevalence of specific diseases. Five (T1–T5) of the eight hosts were used for internal training and testing, and the other three (T6–T8) hosts were only used for the external test. At each site, six board-certified radiologists were involved to annotate the segmentation masks for the mediastinal neoplasms. We used a learning-based method (DenseNet121^40^) to crop the lung area from the original 3D images while keeping the aspect ratio of every 2D slice which is 512 × 512. The image slices were then resized to 128 × 128 and normalized to [−1, 1] from Hounsfield units (HU) between −160 and 240. Finally, a total of 27,048 2D image slices from 575 series were used in this dataset, which is all plain CT images. The samples in each internal host were split randomly and subject-wise into training (80%) and test sets (20%), while all the samples in external hosts contributed to test sets. More details are shown in Supplementary Table 1.

#### Brain Midline dataset (M1-M8)

The Brain Midline dataset was built from eight clinical institutions—The 924th Hospital of Chinese PLA Joint Logistics Support Force (M1), Langfang TCM Hospital (M2), The 908th Hospital of Chinese PLA Joint Logistics Support Force (M3), Sinopharm Gezhouba Central Hospital (M4), Chinese PLA General Hospital (M5), The 921st Hospital of the Joint Logistics Support Force of the Chinese People’s Liberation Army (M6), The 927th Hospital of the Joint Logistics Support Force of the Chinese People’s Liberation Army (M7), The 922nd Hospital of the Joint Logistics Support Force of the Chinese People’s Liberation Army (M8). At each site, all the brain CT scans captured in the institution during a randomly selected period were collected to build the dataset(M1: Jul. 4th, 2020 to Oct. 19th, 2020; M2: Jul. 17th, 2020 to Nov. 9th, 2020; M3: Feb. 28th, 2021 to May 5th, 2021; M4: Feb. 19th, 2018 to Apr. 11th, 2018; M5: Mar. 3rd, 2019 to Apr. 28th, 2019; M6: Nov. 15th, 2020 to Jul. 21st, 2021; M7: Nov. 10th, 2020 to Apr. 29, 2021; M8: Nov. 5th, 2019 to May 29th, 2021). The inclusion periods were random and different among sites to mimic a popular clinical application that developed models are updated gradually over a long time. The original head CT images were 3D cubes, and only the 2D slices in the axial plane from the top of the brain to the bottom of the temporal lobe were selected, which contains the majority of the brain area that has a clear midline. The data process, model training, and testing were conducted inside each site. A total of 12,281 2D slices from 599 patients were selected. The 2D images were then normalized to [−1, 1] from HU between −45 and 115. The original 512 × 512 slices were resized to 256 × 256, annotated by two clinicians, reviewed by another experienced radiologist in each site, and then resized again to 128 × 128 before training for a better computation cost. Annotators marked the brain midline with a polyline. Then the polyline result was transformed to a segmentation mask using a line width of 10 pixels. The samples in each internal institution were split randomly and subject-wise into training (80%) and test sets (20%). More details are shown in Supplementary Table 1.

### Relay Learning framework

Relay Learning is a secure de-connection multi-site deep learning framework compatible with various deep neural models for different medical tasks. It benefits from large-scale data in multiple data hosts, while still providing physical security to data privacy. In each Relay Learning instance, the data ${\{{{{{\mathcal{D}}}}}_{i}\}}_{i = 1}^{m}$ in hosts ${\{{{{{\mathcal{H}}}}}_{i}\}}_{i = 1}^{m}$, where *m* is the number of hosts, are processed in a sequence. Only the deep model has the authority to access in and out of each host without the requirement for simultaneous connection via the Internet.

**Fig. 5:**
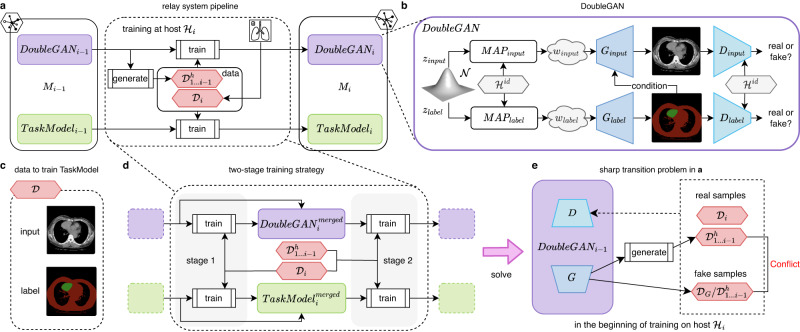
Framework of Relay Learning. **a** Regular training pipeline of the relay system at each host. The model *M*_*i*−1_ transported from host ${{{{\mathcal{H}}}}}_{i-1}$ consists of two components: *DoubleGAN*_*i*−1_ to memorize previous data distribution and *TaskModel*_*i*−1_ to perform specific tasks (medical image segmentation here). At host ${{{{\mathcal{H}}}}}_{i}$, these two components are trained by the real data ${{{{\mathcal{D}}}}}_{i}$ from ${{{{\mathcal{H}}}}}_{i}$ and the heritage data ${{{{\mathcal{D}}}}}_{1...i-1}^{h}$ generated by *DoubleGAN*_*i*−1_. Then, these two updated components build the model *M*_*i*_ and are carried to the next host. **b** The structure of DoubleGAN, which consists of InputGAN (upper half) and LabelGAN (lower half). Each GAN involves a mapping network *MAP* to project input noise to the latent space *ω*, a generator *G* to generate images, and a discriminator *D* to judge whether the generated images are real or fake. We use host identifier ${{{{\mathcal{H}}}}}^{id}$ as a condition in *MAP* and *D*, which instructs DoubleGAN to learn the data distribution of specific hosts. The InputGAN is also conditioned by the generation of LabelGAN, which forces the paired generation to align. **c** The data ${{{\mathcal{D}}}}$ consists of both input and label images, which can train the *TaskModel*. **e** There exists a sharp transition problem of *DoubleGAN* in the regular training pipeline in (**a**). In the begining of training on host ${{{{\mathcal{H}}}}}_{i}$, the generator of *DoubleGAN*_*i*−1_ generates data ${{{{\mathcal{H}}}}}_{1...i-1}$ as real samples together with ${{{{\mathcal{D}}}}}_{i}$. However, ${{{{\mathcal{H}}}}}_{1...i-1}$ is also considered as the fake samples when training *DoubleGAN*_*i*−1_ because it is the output of the generator. This is a conflict and may destabilize the training of *DoubleGAN*_*i*−1_. **d** Therefore, we use a two-stage training strategy to fix this problem. In stage 1, *DoubleGAN*_*i*−1_ and *TaskModel*_*i*−1_ are only trained on data ${{{{\mathcal{D}}}}}_{i}$. Then, we merge the fine-tuned models with the original models to build $DoubleGA{N}_{i}^{merged}$ and $TaskMode{l}_{i}^{merged}$. In stage 2, the normal training as that in (**a**) is conducted using both ${{{{\mathcal{D}}}}}_{i}$ and ${{{{\mathcal{D}}}}}_{1...i-1}^{h}$. See the Methods section for details.

The pipeline shown in Fig. 5a is similar to the sequential finetuning strategy. A model is trained on host ${{{{\mathcal{H}}}}}_{1}$, finetuned on host ${{{{\mathcal{H}}}}}_{2}$, then ${{{{\mathcal{H}}}}}_{3}$... However, in sequential baseline, previous knowledge is easily forgotten when finetuning on new hosts. The core of Relay Learning is to facilitate the task model with a DoubleGAN-based relay system that is capable to replay the previous knowledge. DoubleGAN consists of two generative adversarial networks (GAN), which model the images and labels separately. The objective of DoubleGAN in ${{{{\mathcal{H}}}}}_{i}$ is to create virtual heritage data ${{{{\mathcal{D}}}}}_{1...i-1}^{h}$ that shares the same distribution as previously seen data ${{{{\mathcal{D}}}}}_{1...i-1}$, and use this virtual data to keep the previously learned knowledge in the new host. Data in ${{{{\mathcal{D}}}}}_{1...i-1}^{h}$ keeps the style and distribution of that in ${{{{\mathcal{D}}}}}_{1...i-1}$, but samples are all individually different, thus called the heritage data. Like a sequential finetuning pipeline, the task model and DoubleGAN are updated in each host and carried to the next host in a sequence until the last host is enrolled. The implementation details of the model and training settings can be seen in the Supplementary Notes section.

### DoubleGAN-based relay system

The key objective in Relay Learning is training the task model in new hosts without forgetting the old knowledge learned in previous hosts. However, the restriction of data privacy means storing previous data directly is forbidden. With the rapid development of advanced generative approaches that can form the joint probability distribution of data, we found its ability in creating synthetic data is useful in this situation. Therefore, we propose a DoubleGAN-based relay system as the main component of the Relay Learning framework. DoubleGAN comprises two GANs: InputGAN for input generation and LabelGAN for label generation (Fig. 5b), e.g., input images and segmentation masks in a medical image segmentation task. The aim of DoubleGAN in ${{{{\mathcal{H}}}}}_{i}$ is to recall the knowledge in the training trajectory, generate the heritage data ${{{{\mathcal{D}}}}}_{1...i-1}^{h}$, and utilize it together with the current data ${{{{\mathcal{D}}}}}_{i}$ to train the task model and DoubleGAN itself. The main structures of InputGAN and LabelGAN are based on StyleGAN2-ADA^41^: Each GAN has a mapping network *MAP*_*input*_ or *MAP*_*label*_ that transits the sampled noise *z*_*input*_ or *z*_*label*_ from the standard Gaussian distribution ${{{\mathcal{N}}}}$ to a style code *w*_*input*_ or *w*_*label*_ in a high-dimensional style space, a generator *G*_*input*_ or *G*_*label*_ that generates the task inputs according to the transformed style code *w*_*input*_ or *w*_*label*_, and a discriminator *D*_*input*_ or *D*_*label*_ that judges whether the generation is similar to the real data or not.

To better control DoubleGAN in this multi-site environment, we use conditioning methods to improve its ability. The generated heritage data is conditioned in two aspects: (1) the mapping networks and discriminators are hard-coded with a ${{{{\mathcal{H}}}}}^{id}$ condition that identifies various data distributions across hosts via class-conditioning^41^, which enables DoubleGAN to generate data of specific host; (2) *G*_*input*_ is conditioned by the generation of *G*_*label*_ using SPADE^42^, which establishes the paired relationship between the generated inputs and labels on the image level.

### Two-stage strategy

In traditional sequential training or fine-tuning procedure, the pre-trained model will be directly trained on new data. At first, our relay system followed this pipeline as shown in Fig. 5a. However, we found that there is a sharp transition problem for the training of DoubleGAN in Relay Learning as shown in Fig. 5e. The responsibility of discriminators in DoubleGAN is to tell whether the results of generators are real data or fake data. At the beginning of training on ${{{{\mathcal{H}}}}}_{i}$, we use *DoubleGAN*_*i*−1_ to generate heritage data ${{{{\mathcal{D}}}}}_{1...i-1}^{h}$. This data contains the data distribution information in previous sites ${{{{\mathcal{H}}}}}_{1...i-1}$. It will be used as real samples together with the current data ${{{{\mathcal{D}}}}}_{i}$ from ${{{{\mathcal{H}}}}}_{i}$, to keep training *DoubleGAN*_*i*−1_. However, at the beginning of the training procedure, the same data ${{{{\mathcal{D}}}}}_{1...i-1}^{h}$ is also the output of the generator of *DoubleGAN*_*i*−1_, which should be used as fake samples to train the discriminator. This is a conflict and a sharp transition of the expected judgment, which will destabilize the discriminator training, and consequently influence the generators.

Therefore, we use a two-stage strategy in ${{{{\mathcal{H}}}}}_{i}$ to solve this problem as shown in Fig. 5d. In the first stage, *DoubleGAN*_*i*−1_ and the task model are only trained on current data ${{{{\mathcal{D}}}}}_{i}$ like a fine-tuning routine, resulting in $DoubleGA{N}_{i}^{f}$, which is only capable to generate ${{{{\mathcal{D}}}}}_{i}^{h}$. Then, this fine-tuned model is weighted-merged with the original *DoubleGAN*_*i*−1_ as follows:

1 $${\theta }_{DoubleGA{N}_{i}^{merged}}=\frac{{\bar{b}}_{i-1}}{{\bar{b}}_{i-1}+{b}_{i}}{\theta }_{DoubleGA{N}_{i-1}}+\frac{{b}_{i}}{{\bar{b}}_{i-1}+{b}_{i}}{\theta }_{DoubleGA{N}_{i}^{f}}$$

where *θ* is the model parameter, *b*_*i*_ is the number of samples in ${{{{\mathcal{D}}}}}_{i}$, and ${\bar{b}}_{i}=\mathop{\sum }\nolimits_{j = 1}^{i}{b}_{j}$. This parameter merging combines the ability in *DoubleGAN*_*i*−1_ and $DoubleGA{N}_{i}^{f}$. Then, the second stage is performed using both ${{{{\mathcal{D}}}}}_{1...i-1}^{h}$ and ${{{{\mathcal{D}}}}}_{i}$ to train $DoubleGA{N}_{i}^{merged}$, finally resulting in updated *DoubleGAN*_*i*_. Similarly as DoubleGAN, the task model is also trained using this two-stage strategy. This strategy is not applied in ${{{{\mathcal{H}}}}}_{1}$ as the model is trained from scratch there.

### Pseudo labels

In complicated medical tasks, e.g., pixel-wise lesion segmentation tasks on medical images, the generated labels, i.e., lesion segmentation masks, tend to model the real distribution of the abnormalities in the human body, which may be markedly unbalanced with numerous negative samples. In such cases, the training of InputGAN that is conditioned on lesion masks may be unstable. Therefore, we introduce pseudo labels applied to the original labels that assist the LabelGAN to produce additional information. Pseudo labels can be in various forms depending on the characteristics of the task. In our mediastinum tumor and brain midline experiments, we clip the input image according to HU threshold ranges that differ the background and tissue in segmentation tasks as the pseudo label.

### Evaluation metrics and statistical analysis

Following the common practice in voxel-level medical image tasks, we mainly used two evaluation metrics in the experiments, Hausdorff Distance (HD) for the Brain Midline dataset and Dice similarity coefficient (Dice) for Mediastinum Tumor and Retina Fundus datasets. The calculation for Hausdorff Distance is:

2 $$HD(P,L)=\max \{\mathop{\max }\limits_{p\in P}\mathop{\min }\limits_{l\in L}dist(p,l),\mathop{\max }\limits_{l\in L}\mathop{\min }\limits_{p\in P}dist(l,p)\}$$

where *P* and *L* are the boundary point sets in the prediction and label masks, in which *p* and *l* are specific points. *dist*(*x*, *y*) computes the Euclidean distance between two points *x* and *y*. To eliminate the influence of outliers, we used 95% HD which is based on the 95th percentile in the computation of maximum and minimum distances between *P* and *L*. The Dice score is computed as:

3 $$Dice=\frac{2{{{\rm{TP}}}}}{2{{{\rm{TP}}}}+{{{\rm{FP}}}}+{{{\rm{FN}}}}}$$

where TP, FP, and FN indicate true positive, false positive, and false negative points between the prediction and the label masks.

The 95% CI of the mean metrics in all experiments was estimated with all the values in each test experiment. Statistical analysis in Fig. 2c, g, Fig. 3c, g, and Fig. 4c, f were tested using ordinary ANOVA test and Holm-Šidák’s multiple comparisons test, with a single pooled variance. Statistical analysis in Fig. 2d, h, and Fig. 3d, h were tested using ordinary one-way ANOVA test and Dunnett’s multiple comparisons test, with a single pooled variance. Statistical analysis in Fig. 2e, i, Fig. 3e, i, and Fig. 4d, g were tested using ordinary two-way ANOVA and Tukey’s multiple comparisons test, with individual variances computed for each comparison. No statistical methods were used to predetermine the sample size. Measurements were tested on the same test sets using different models in each experiment.

### Human subject data

The study on the public Retina Fundus dataset follows the data usage principle of the original provider. The study on the Mediastinum Tumor dataset was approved by the Ethics Committee of the National Center for Respiratory Medicine/The First Affiliated Hospital of Guangzhou Medical University. The study on the Brain Midline dataset was approved by the Ethics Committee of Chinese PLA General Hospital/Chinese PLA Medical School. Informed consent was waived for retrospectively collected medical images, which were anonymized before data processing.

### Supplementary information

Supplementary Information

## Data Availability

The whole public Retina Fundus dataset was released in previous research and can be found at https://drive.google.com/file/d/1p33nsWQaiZMAgsruDoJLyatoq5XAH-TH/viewand https://www.kaggle.com/datasets/sshikamaru/glaucoma-detection. The Mediastinum Tumor dataset can be accessed by contacting our first or corresponding authors. Only requests with appropriate research purposes can be authorized. Due to privacy regulations, we will not release the Brain Midline dataset.

## Code availability

The entire training, validation, and application code in Relay Learning are released on https://github.com/MeteorsHub/RelayLearning.

## References

[1] Price WN, Cohen IG (2019). Privacy in the age of medical big data. Nat. Med..

[2] Rajpurkar P, Chen E, Banerjee O, Topol EJ (2022). Ai in health and medicine. Nat. Med..

[3] Campanella G (2019). Clinical-grade computational pathology using weakly supervised deep learning on whole slide images. Nat. Med..

[4] Lu MY (2021). Ai-based pathology predicts origins for cancers of unknown primary. Nature.

[5] Yao X (2021). Artificial intelligence–enabled electrocardiograms for identification of patients with low ejection fraction: a pragmatic, randomized clinical trial. Nat. Med..

[6] Chilamkurthy S (2018). Deep learning algorithms for detection of critical findings in head ct scans: a retrospective study. Lancet.

[7] Cen L-P (2021). Automatic detection of 39 fundus diseases and conditions in retinal photographs using deep neural networks. Nat. Commun..

[8] Ardila D (2019). End-to-end lung cancer screening with three-dimensional deep learning on low-dose chest computed tomography. Nat. Med..

[9] Konečny`, J. et al. Federated learning: strategies for improving communication efficiency. *arXiv preprint arXiv:1610.05492* (2016).

[10] Kaissis GA, Makowski MR, Rückert D, Braren RF (2020). Secure, privacy-preserving and federated machine learning in medical imaging. Nat. Mach. Intell..

[11] Warnat-Herresthal S (2021). Swarm learning for decentralized and confidential clinical machine learning. Nature.

[12] Li Z, Hoiem D (2017). Learning without forgetting. IEEE Trans. Pattern Anal. Mach. Intell..

[13] Zhang K (2021). Deep-learning models for the detection and incidence prediction of chronic kidney disease and type 2 diabetes from retinal fundus images. Nat. Biomed. Eng..

[14] Garway-Heath DF, Ruben ST, Viswanathan A, Hitchings RA (1998). Vertical cup/disc ratio in relation to optic disc size: its value in the assessment of the glaucoma suspect. Br. J. Ophthalmol..

[15] Sivaswamy J (2015). A comprehensive retinal image dataset for the assessment of glaucoma from the optic nerve head analysis. JSM Biomed. Imaging Data Papers.

[16] Fumero, F., Alayón, S., Sanchez, J. L., Sigut, J. & Gonzalez-Hernandez, M. Rim-one: an open retinal image database for optic nerve evaluation. In *2011 24th international symposium on computer-based medical systems (CBMS)*, 1–6 (IEEE, 2011).

[17] Orlando JI (2020). Refuge challenge: a unified framework for evaluating automated methods for glaucoma assessment from fundus photographs. Med. Image Anal..

[18] Zhang, Z. et al. Origa-light: An online retinal fundus image database for glaucoma analysis and research. In *2010 Annual international conference of the IEEE engineering in medicine and biology*, 3065–3068 (IEEE, 2010). 10.1109/IEMBS.2010.5626137 21095735

[19] Araki T (2015). Anterior mediastinal masses in the framingham heart study: prevalence and ct image characteristics. Eur. J. Radiol. Open.

[20] Duwe BV, Sterman DH, Musani AI (2005). Tumors of the mediastinum. Chest.

[21] De Lange M (2021). A continual learning survey: Defying forgetting in classification tasks. IEEE Trans. Pattern Anal. Mach. Intell..

[22] Liao, C.-C. et al. Brain midline shift measurement and its automation: a review of techniques and algorithms. *Int. J. Biomed. Imaging* **2018** (2018). 10.1155/2018/4303161 PMC5925103 29849536

[23] Goodfellow, I. et al. Generative adversarial nets. *Adv. Neural Inform. Process. Syst.* **27** (2014).

[24] Zhu, J.-Y., Park, T., Isola, P. & Efros, A. A. Unpaired image-to-image translation using cycle-consistent adversarial networks. In *Proc. IEEE International Conference on Computer Vision*, 2223–2232 (2017).

[25] Karras, T., Laine, S. & Aila, T. A style-based generator architecture for generative adversarial networks. In *Proc. IEEE/CVF Conference on Computer Vision and Pattern Recognition*, 4401–4410 (2019). 10.1109/TPAMI.2020.2970919 32012000

[26] Zhang, Y. et al. Datasetgan: efficient labeled data factory with minimal human effort. In *Proc. IEEE/CVF Conference on Computer Vision and Pattern Recognition*, 10145–10155 (2021).

[27] Kim BN, Dolz J, Jodoin P-M, Desrosiers C (2021). Privacy-net: an adversarial approach for identity-obfuscated segmentation of medical images. IEEE Trans. Med. Imaging.

[28] Kim T, Yang J (2019). Latent-space-level image anonymization with adversarial protector networks. IEEE Access.

[29] Wu Y, Yang F, Xu Y, Ling H (2019). Privacy-protective-gan for privacy preserving face de-identification. J. Comput. Sci. Technol..

[30] Perkonigg M (2021). Dynamic memory to alleviate catastrophic forgetting in continual learning with medical imaging. Nat. Commun..

[31] Kiyasseh D, Zhu T, Clifton D (2021). A clinical deep learning framework for continually learning from cardiac signals across diseases, time, modalities, and institutions. Nat. Commun..

[32] Zeng G, Chen Y, Cui B, Yu S (2019). Continual learning of context-dependent processing in neural networks. Nat. Mach. Intell..

[33] Van de Ven GM, Siegelmann HT, Tolias AS (2020). Brain-inspired replay for continual learning with artificial neural networks. Nat. Commun..

[34] Fredrikson, M., Jha, S. & Ristenpart, T. Model inversion attacks that exploit confidence information and basic countermeasures. In *Proc. 22nd ACM SIGSAC Conference on Computer and Communications Security*, 1322–1333 (2015).

[35] Wang, Z. et al. Beyond inferring class representatives: user-level privacy leakage from federated learning. In *IEEE INFOCOM 2019-IEEE Conference on Computer Communications*, 2512–2520 (IEEE, 2019).

[36] Bentzen HB (2021). Remove obstacles to sharing health data with researchers outside of the european union. Nat. Med..

[37] Wang S (2020). Dofe: Domain-oriented feature embedding for generalizable fundus image segmentation on unseen datasets. IEEE Trans. Med. Imaging.

[38] Liu, Q., Chen, C., Qin, J., Dou, Q. & Heng, P.-A. Feddg: Federated domain generalization on medical image segmentation via episodic learning in continuous frequency space. In *Proc. IEEE/CVF Conference on Computer Vision and Pattern Recognition*, 1013–1023 (2021).

[39] Tang R (2023). Pan-mediastinal neoplasm diagnosis via nationwide federated learning: a multicentre cohort study. Lancet Digital Health.

[40] Huang, G., Liu, Z., Van Der Maaten, L. & Weinberger, K. Q. Densely connected convolutional networks. In *Proc. IEEE Conference on Computer Vision and Pattern Recognition*, 4700–4708 (2017).

[41] Karras T (2020). Training generative adversarial networks with limited data. Adv. Neural Inf. Process. Syst..

[42] Park, T., Liu, M.-Y., Wang, T.-C. & Zhu, J.-Y. Semantic image synthesis with spatially-adaptive normalization. In *Proc. IEEE/CVF Conference on Computer Vision and Pattern Recognition*, 2337–2346 (2019).

