# Supplementary material for: Relay learning: a physically secure framework for clinical multi-site deep learning
Source: NPJ Digit Med. 2023 Nov 4;6:204. doi: 10.1038/s41746-023-00934-4 (PMC10625523; doi:10.1038/s41746-023-00934-4)
Supplement: Supplementary file 1 — Supplementary Information [file 41746_2023_934_MOESM1_ESM.pdf]

Supplementary Information - Relay Learning: a Physically Secure Framework for Clinical Multi-site Deep Learning

Zi-Hao Bo[1,3], Yuchen Guo[3*], Jinhao Lyu[2], Hengrui Liang[4], Jianxing He[4], Shijie Deng[5], Feng Xu[1,3*], Xin Lou[2*] and Qionghai Dai[3,6*]

[1]School of Software, Tsinghua University, Beijing, China.
[2]Department of Radiology, Chinese PLA General Hospital / Chinese PLA Medical School, Beijing, China.
[3]BNRist, Tsinghua University, Beijing, China.
[4]Department of Thoracic Oncology and Surgery, China State Key Laboratory of Respiratory Disease & National Clinical Research Center for Respiratory Disease, the First Affiliated Hospital of Guangzhou Medical University, Guangzhou, China.
[5]Department of Radiology, the 921st Hospital of Chinese PLA, Changsha, China.
[6]Department of Automation, Tsinghua University, Beijing, China.

*Corresponding author(s). E-mail(s): Yuchen.w.guo@gmail.com; feng-xu@tsinghua.edu.cn; louxin@301hospital.com.cn; qhdai@tsinghua.edu.cn;

Supplementary Results

Evaluation details. The details of the three datasets used in this research are shown in Supplementary Table 1. We also show experiment details of different methods evaluated on test sets from different clinical sites in Supplementary Table 2, Supplementary Table 4, and Supplementary Table 5. Experiment details of Federated Learning and Swarm Learning are shown in Supplementary Table 6. The statistical analysis of Relay Learning and other methods on the three datasets is shown in Supplementary Table 7. To show the effectiveness of our DoubleGAN-based relay system, we include a visualization of the generated heritage data and real data samples on the Retina Fundus dataset in Supplementary Fig. 2.

Full permutation analysis. Besides the three episodes in the Results section, we also evaluated all the permutations of training host order on the Retina Fundus dataset, which results in 24 permutations given four internal hosts. The result is shown in Supplementary Table 8 and Supplementary Fig. 1. Relay Learning performed uniformly well in all the permutations. It was close to central learning on internal test sets and even better on the external test set.

Compared to compatible methods. Besides the sequential learning methods, we also compared our Relay Learning to some compatible methods in continual learning. Although many continual learning approaches leak data privacy due to the storage of original data, there are a few regularization-based methods that are compatible with our setting. However, without data reuse, they are still immature in complicated pixel-level tasks. First, we compared SI (synaptic intelligence) [1], a method that penalizes the modification of important parameters in new hosts. Then, we compared OWM (orthogonal weights modification) [2], which tries to update parameters in the orthogonal space of previous data. We compared these two methods with sequential learning and our Relay Learning approaches on the Retina Fundus dataset, which is shown in Supplementary Table 9 and Supplementary Fig. 3. In our pixel-level experiment, SI and OWM both performed weakly and almost the same as sequential learning. The reason may be that

simple weight regularization is only capable in easy classification tasks and not enough for complicated tasks.

Analysis of two-stage strategy. We also investigated the utility of our two-stage training strategy in the relay system. If without the two-stage strategy, we directly fine-tuned the DoubleGAN and the task model in new hosts, which is called the one-stage strategy. The result performed on the Retina Fundus dataset is shown in Supplementary Table 9 and Supplementary Fig. 3. Because of the sharp transition problem described in the Methods section, the one-stage method was worse than the two-stage method by a large margin.

Supplementary Notes

Training details. In multi-site Relay Learning and other compared methods, we trained the network with a batch size of 64 on two GPUs. We kept training task models in each institution until the loss stabilizes, which varies from about one to three million samples depending on different datasets. We used Adam [3] optimizer to train the task model, with a learning rate of 0.001.

Task model. We used U-Net [4] structure as our task model in all of the three datasets, with a ResNet18 [5] as the backbone encoder which was pre-trained on ImageNet [6]. The decoder was composed of several transpose convolution layers. The output channel number in the decoder of U-Net equals the number of task label classes, which was two in the Mediastinum Tumor and Brain Midline datasets, and three in the Retina Fundus dataset. We used LeakyRelu [7] as the activation layers, and Exp loss [8] as the loss function of the task model, which was composed of Dice loss and cross-entropy loss, whose weights were 0.8 and 0.2 respectively.

Relay Learning. The DoubleGAN-based relay system in Relay Learning consists of an InputGAN and a LabelGAN, which were both based on StyleGAN2-ADA [9]. The dimensions of z_{input} and z_{label} were 128 and 64, while that of w_{input} and w_{label} were 256 and 128. The numbers of mapping layers were 4 and 2 for InputGAN and LabelGAN. Before feeding into LabelGAN, the segmentation label was mapped to (-1, 1). We also

added a truncated Gaussian noise with a sigma of 0.02 to the real label to stabilize the training procedure. The style mixing probability in DoubleGAN was set to 0.8. In the relay system, we kept the training batch size unchanged, with half the samples replaced by heritage data, The task ids in the heritage data were uniformly selected from previously seen institutions. The generated samples were augmented in brightness and geometry. We also added a distillation loss to the task model when training with heritage data, whose weight was 0.5. The adaptive augmentation introduced in StyleGAN2-ADA in both InputGAN and LabelGAN included image flip, rotation, isotropic and anisotropic scaling, brightness and contrast adjustment, luma flip, additive noise, and so on. The targets of adaptive augmentation were 0.7 and 0.8 in InputGAN and LabelGAN, while the probability was cut in half when transiting to a new institution. The optimizer was also Adam but with a learning rate of 0.002 in the two GANs. The absolute value of gradients in the relay system was clipped under 100,000 and set to 0 when NaN happens to avoid training failure. In the training of InputGAN, we used the real label map as the SPADE [9] condition to InputGAN, while when generating, the output of LabelGAN was used as the condition. Pseudo labels were added to the DoubleGAN training. In the Mediastinum Tumor dataset, we added a background mask for pixels that have Hounsfield units (HU) less than -500. In the Brain Midline dataset, we added a background mask for pixels that have HU less than -38 and contain no midline annotations.

Other methods The sequential and central learning strategies used the same U-Net structure task model and followed the same training details described above, except that the central learning includes only one simulated central host that combines the data from all the hosts. The sequential methods were directly fine-tuned on new hosts. We simulated Federated Learning and Swarm Learning at a virtual central machine on the Retina Fundus dataset and the Mediastinum Tumor dataset. Separate models for each site were trained on data from their own site. A central model was synchronized every training epoch from all separate models using parameter aggregation. The parameter aggregation in Federated Learning was weighted average by the number of training samples at each site following FedAvg [10]. The parameter aggregation in Swarm Learning was plain

average without weighting, following [11]. We implemented SI and OWM methods in our framework. The hyper-parameter c in SI was set to 0.5. In the OWM method, we implemented batch-normalization and transposed convolution layers by ourselves according to the description in the paper, because its original network only consists of three simple convolution layers and three fully-connected layers, which is incompatible with our segmentation model.

References

[1] Zenke, F., Poole, B., Ganguli, S.: Continual learning through synaptic intelligence. In: International Conference on Machine Learning, pp. 3987–3995 (2017). PMLR

[2] Zeng, G., Chen, Y., Cui, B., Yu, S.: Continual learning of context-dependent processing in neural networks. Nature Machine Intelligence **1**(8), 364–372 (2019)

[3] Kingma, D.P., Ba, J.: Adam: A method for stochastic optimization. arXiv preprint arXiv:1412.6980 (2014)

[4] Ronneberger, O., Fischer, P., Brox, T.: U-net: Convolutional networks for biomedical image segmentation. In: Medical Image Computing and Computer-Assisted Intervention–MICCAI 2015: 18th International Conference, Munich, Germany, October 5-9, 2015, Proceedings, Part III 18, pp. 234–241 (2015). Springer

[5] He, K., Zhang, X., Ren, S., Sun, J.: Deep residual learning for image recognition. In: Proceedings of the IEEE Conference on Computer Vision and Pattern Recognition, pp. 770–778 (2016)

[6] Russakovsky, O., Deng, J., Su, H., Krause, J., Satheesh, S., Ma, S., Huang, Z., Karpathy, A., Khosla, A., Bernstein, M., *et al.*: Imagenet large scale visual recognition challenge. International journal of computer vision **115**, 211–252 (2015)

[7] Maas, A.L., Hannun, A.Y., Ng, A.Y., *et al.*: Rectifier nonlinearities improve neural network acoustic models. In: Proc. Icml, vol. 30, p. 3 (2013). Atlanta, GA

[8] Wong, K.C., Moradi, M., Tang, H., Syeda-Mahmood, T.: 3d segmentation with exponential logarithmic loss for highly unbalanced object sizes. In: Medical Image Computing and Computer Assisted Intervention–MICCAI 2018: 21st International Conference, Granada, Spain, September 16-20, 2018, Proceedings, Part III 11, pp. 612–619 (2018). Springer

[9] Park, T., Liu, M.-Y., Wang, T.-C., Zhu, J.-Y.: Semantic image synthesis with spatially-adaptive normalization. In: Proceedings of the IEEE/CVF Conference on Computer Vision and Pattern Recognition, pp. 2337–2346 (2019)

[10] McMahan, B., Moore, E., Ramage, D., Hampson, S., y Arcas, B.A.: Communication-efficient learning of deep networks from decentralized data. In: Artificial Intelligence and Statistics, pp. 1273–1282 (2017). PMLR

[11] Warnat-Herresthal, S., Schultze, H., Shastry, K.L., Manamohan, S., Mukherjee, S., Garg, V., Sarveswara, R., Händler, K., Pickkers, P., Aziz, N.A., *et al.*: Swarm learning for decentralized and confidential clinical machine learning. Nature **594**(7862), 265–270 (2021)

Supplementary Table 1 Statistics of the three datasets in evaluation.

dataset	train set		test set		total	
	#series	#slices	#series	#slices	#series	#slices
F1	-	50	-	51	-	101
F2	-	99	-	60	-	159
F3	-	320	-	80	-	400
F4	-	320	-	80	-	400
F5	-	-	-	649	-	649
total	-	789	-	920	-	1,709
T1	90	3,288	23	881	113	4,169
T2	56	2,640	14	634	70	3,274
T3	52	2,673	14	724	66	3,397
T4	32	1,478	9	441	41	1,919
T5	55	2,532	14	631	69	3,163
T6	-	-	17	445	17	445
T7	-	-	16	874	16	874
T8	-	-	183	9,807	183	9,807
total	285	12,611	290	14,437	575	27,048
M1	77	2,442	20	634	97	3,076
M2	64	2,584	17	691	81	3,275
M3	62	1,424	16	360	78	1,784
M4	29	612	8	182	37	794
M5	76	1,751	20	460	96	2,211
M6	-	-	125	911	125	911
M7	-	-	20	64	20	64
M8	-	-	65	166	65	166
total	308	8,813	291	3,468	599	12,281

F1-F5: Retina Fundus dataset; T1-T8: Mediastinum Tumor dataset; M1-M8: Brain Midline dataset; All the numbers of series are the same as the numbers of patients. #: number of.

Supplementary Table 2 Evaluation details on Retina Fundus dataset (OC/OC averaged).

	local					sequential				Relay				central
	1	2	3	4	avg.	ep.1	ep.2	ep.3	avg.	ep.1	ep.2	ep.3	avg.	
F1-test	0.797<br>0.761-0.832	0.702<br>0.670-0.734	0.724<br>0.687-0.761	0.385<br>0.325-0.444	0.652<br>0.622-0.682	0.349<br>0.298-0.400	0.845<br>0.822-0.868	0.838<br>0.816-0.861	0.677<br>0.635-0.719	0.787<br>0.763-0.812	0.822<br>0.800-0.844	0.859<br>0.841-0.876	0.823<br>0.810-0.836	0.836<br>0.808-0.863
F2-test	0.526<br>0.461-0.590	0.754<br>0.714-0.793	0.620<br>0.567-0.672	0.342<br>0.264-0.421	0.560<br>0.525-0.595	0.250<br>0.177-0.323	0.632<br>0.581-0.683	0.581<br>0.515-0.647	0.488<br>0.444-0.532	0.751<br>0.715-0.787	0.783<br>0.750-0.816	0.713<br>0.663-0.763	0.749<br>0.726-0.772	0.786<br>0.750-0.821
F3-test	0.703<br>0.672-0.735	0.737<br>0.714-0.760	0.879<br>0.863-0.895	0.794<br>0.771-0.817	0.778<br>0.764-0.792	0.818<br>0.794-0.841	0.811<br>0.789-0.833	0.787<br>0.762-0.813	0.805<br>0.792-0.819	0.873<br>0.858-0.888	0.855<br>0.840-0.869	0.858<br>0.844-0.872	0.862<br>0.854-0.870	0.887<br>0.871-0.903
F4-test	0.139<br>0.117-0.161	0.292<br>0.266-0.318	0.642<br>0.614-0.671	0.886<br>0.873-0.898	0.490<br>0.456-0.524	0.884<br>0.868-0.899	0.319<br>0.288-0.350	0.244<br>0.218-0.271	0.482<br>0.443-0.521	0.880<br>0.867-0.894	0.822<br>0.802-0.841	0.879<br>0.866-0.893	0.860<br>0.851-0.870	0.892<br>0.878-0.905
int. avg.	0.515<br>0.479-0.551	0.603<br>0.575-0.631	0.722<br>0.702-0.743	0.644<br>0.608-0.680	0.621<br>0.605-0.637	0.623<br>0.585-0.662	0.632<br>0.602-0.663	0.591<br>0.557-0.625	0.616<br>0.596-0.635	0.832<br>0.819-0.845	0.823<br>0.812-0.834	0.832<br>0.818-0.847	0.829<br>0.822-0.837	0.856<br>0.844-0.868
F5-test/<br>ext. avg.	0.578<br>0.564-0.591	0.631<br>0.620-0.642	0.505<br>0.493-0.517	0.268<br>0.252-0.283	0.495<br>0.487-0.504	0.306<br>0.290-0.322	0.698<br>0.688-0.708	0.628<br>0.615-0.641	0.544<br>0.533-0.555	0.747<br>0.736-0.758	0.765<br>0.756-0.773	0.716<br>0.703-0.729	0.742<br>0.736-0.749	0.583<br>0.566-0.600
avg.	0.559<br>0.545-0.574	0.623<br>0.611-0.634	0.569<br>0.557-0.581	0.378<br>0.360-0.397	0.532<br>0.525-0.540	0.399<br>0.381-0.418	0.679<br>0.667-0.690	0.617<br>0.603-0.631	0.565<br>0.555-0.575	0.772<br>0.763-0.781	0.782<br>0.775-0.789	0.750<br>0.739-0.761	0.768<br>0.763-0.773	0.663<br>0.649-0.678

Results are Dice values (higher is better) with 95% CI. int.: internal (F1-F4) test sets; ext.: external (F5) test set; ep.: episode; avg.: average.

Supplementary Table 3 Evaluation details on Retina Fundus dataset (OC/OD separated).

OC	local 1	local 2	local 3	local 4	local avg.	sequential ep.1	sequential ep.2	sequential ep.3	sequential avg.	Relay ep.1	Relay ep.2	Relay ep.3	Relay avg.	central
F1-test	0.779<br>0.717-0.840	0.730<br>0.694-0.765	0.721<br>0.679-0.764	0.372<br>0.301-0.444	0.651<br>0.616-0.685	0.148<br>0.091-0.205	0.308<br>0.271-0.344	0.575<br>0.492-0.657	0.349<br>0.308-0.390	0.779<br>0.746-0.812	0.814<br>0.790-0.839	0.681<br>0.607-0.755	0.763<br>0.735-0.791	0.853<br>0.815-0.890
F2-test	0.517<br>0.435-0.599	0.703<br>0.644-0.762	0.572<br>0.491-0.654	0.340<br>0.255-0.425	0.533<br>0.492-0.575	0.175<br>0.098-0.251	0.801<br>0.773-0.828	0.136<br>0.107-0.166	0.388<br>0.340-0.437	0.748<br>0.703-0.792	0.833<br>0.813-0.854	0.874<br>0.857-0.891	0.825<br>0.808-0.841	0.772<br>0.726-0.818
F3-test	0.685<br>0.639-0.731	0.751<br>0.722-0.781	0.865<br>0.846-0.884	0.813<br>0.793-0.833	0.779<br>0.762-0.795	0.815<br>0.793-0.838	0.659<br>0.596-0.722	0.763<br>0.724-0.803	0.754<br>0.729-0.778	0.858<br>0.842-0.873	0.753<br>0.714-0.791	0.842<br>0.826-0.858	0.823<br>0.809-0.838	0.874<br>0.855-0.894
F4-test	0.110<br>0.085-0.136	0.240<br>0.204-0.277	0.629<br>0.595-0.662	0.875<br>0.857-0.893	0.464<br>0.427-0.500	0.869<br>0.845-0.893	0.851<br>0.813-0.888	0.844<br>0.809-0.878	0.857<br>0.839-0.874	0.871<br>0.854-0.888	0.834<br>0.807-0.860	0.871<br>0.844-0.899	0.861<br>0.848-0.874	0.883<br>0.866-0.900
int. avg.	0.496<br>0.455-0.537	0.586<br>0.552-0.619	0.703<br>0.677-0.729	0.644<br>0.606-0.681	0.607<br>0.589-0.625	0.564<br>0.518-0.609	0.633<br>0.600-0.666	0.552<br>0.510-0.593	0.583<br>0.559-0.606	0.822<br>0.808-0.837	0.810<br>0.796-0.824	0.821<br>0.801-0.842	0.818<br>0.808-0.827	0.850<br>0.835-0.865
F5-test/<br>ext. avg.	0.552<br>0.535-0.568	0.652<br>0.639-0.665	0.600<br>0.586-0.614	0.310<br>0.289-0.330	0.528<br>0.519-0.538	0.276<br>0.257-0.296	0.701<br>0.689-0.713	0.597<br>0.579-0.614	0.525<br>0.512-0.537	0.760<br>0.748-0.772	0.780<br>0.771-0.790	0.781<br>0.770-0.793	0.774<br>0.768-0.780	0.680<br>0.661-0.698
avg.	0.535<br>0.519-0.552	0.632<br>0.619-0.646	0.631<br>0.618-0.643	0.408<br>0.387-0.428	0.552<br>0.543-0.560	0.361<br>0.340-0.382	0.681<br>0.668-0.694	0.583<br>0.566-0.601	0.542<br>0.531-0.553	0.778<br>0.769-0.788	0.789<br>0.781-0.797	0.793<br>0.783-0.803	0.787<br>0.782-0.792	0.730<br>0.715-0.745

OD	local 1	local 2	local 3	local 4	local avg.	sequential ep.1	sequential ep.2	sequential ep.3	sequential avg.	Relay ep.1	Relay ep.2	Relay ep.3	Relay avg.	central
F1-test	0.815<br>0.793-0.837	0.674<br>0.635-0.714	0.727<br>0.687-0.767	0.397<br>0.337-0.457	0.653<br>0.623-0.683	0.549<br>0.489-0.610	0.330<br>0.300-0.360	0.586<br>0.528-0.645	0.469<br>0.437-0.501	0.796<br>0.771-0.820	0.829<br>0.812-0.847	0.745<br>0.703-0.787	0.794<br>0.777-0.811	0.819<br>0.790-0.847
F2-test	0.534<br>0.473-0.595	0.804<br>0.768-0.839	0.667<br>0.624-0.710	0.344<br>0.266-0.422	0.587<br>0.552-0.622	0.325<br>0.248-0.403	0.822<br>0.799-0.845	0.352<br>0.319-0.385	0.516<br>0.476-0.555	0.754<br>0.717-0.791	0.876<br>0.865-0.888	0.885<br>0.872-0.898	0.846<br>0.832-0.860	0.799<br>0.761-0.836
F3-test	0.722<br>0.693-0.750	0.722<br>0.696-0.749	0.893<br>0.875-0.911	0.775<br>0.741-0.809	0.778<br>0.763-0.793	0.821<br>0.789-0.852	0.605<br>0.550-0.661	0.811<br>0.789-0.833	0.758<br>0.735-0.782	0.889<br>0.871-0.906	0.813<br>0.777-0.849	0.874<br>0.857-0.891	0.863<br>0.849-0.876	0.900<br>0.882-0.918
F4-test	0.168<br>0.145-0.191	0.344<br>0.321-0.367	0.656<br>0.627-0.685	0.896<br>0.886-0.906	0.516<br>0.483-0.549	0.898<br>0.886-0.909	0.838<br>0.822-0.855	0.833<br>0.813-0.853	0.863<br>0.853-0.873	0.889<br>0.876-0.902	0.811<br>0.784-0.837	0.846<br>0.828-0.864	0.855<br>0.844-0.867	0.900<br>0.889-0.912
int. avg.	0.534<br>0.499-0.569	0.620<br>0.593-0.646	0.742<br>0.722-0.761	0.644<br>0.608-0.680	0.635<br>0.619-0.651	0.683<br>0.648-0.718	0.632<br>0.601-0.662	0.630<br>0.600-0.660	0.648<br>0.630-0.667	0.841<br>0.828-0.855	0.836<br>0.825-0.848	0.843<br>0.830-0.857	0.840<br>0.833-0.848	0.862<br>0.850-0.875
F5-test/<br>ext. avg.	0.604<br>0.590-0.617	0.610<br>0.598-0.623	0.409<br>0.396-0.423	0.226<br>0.210-0.241	0.462<br>0.453-0.472	0.335<br>0.317-0.354	0.694<br>0.683-0.705	0.660<br>0.647-0.673	0.563<br>0.552-0.574	0.733<br>0.721-0.746	0.749<br>0.738-0.759	0.651<br>0.632-0.669	0.711<br>0.702-0.719	0.486<br>0.467-0.506
avg.	0.583<br>0.569-0.598	0.613<br>0.601-0.625	0.507<br>0.493-0.522	0.349<br>0.329-0.369	0.513<br>0.505-0.522	0.438<br>0.418-0.457	0.676<br>0.664-0.688	0.651<br>0.638-0.664	0.588<br>0.579-0.598	0.765<br>0.755-0.775	0.774<br>0.766-0.783	0.707<br>0.693-0.722	0.749<br>0.742-0.756	0.597<br>0.579-0.615

Results are Dice values (higher is better) with 95% CI. int.: internal (F1-F4) test sets; ext.: external (F5) test set; ep.: episode; avg.: average.

Supplementary Table 4 Evaluation details on Mediastinum Tumor dataset.

	local						sequential				Relay				central
	1	2	3	4	5	avg.	ep.1	ep.2	ep.3	avg.	ep.1	ep.2	ep.3	avg.	
T1-test	0.403<br>0.270-0.536	0.348<br>0.219-0.478	0.409<br>0.251-0.567	0.345<br>0.194-0.497	0.419<br>0.261-0.577	0.385<br>0.323-0.447	0.374<br>0.213-0.534	0.537<br>0.328-0.746	0.388<br>0.218-0.558	0.409<br>0.322-0.496	0.492<br>0.339-0.645	0.473<br>0.323-0.623	0.504<br>0.352-0.655	0.490<br>0.407-0.573	0.476<br>0.320-0.632
T2-test	0.541<br>0.347-0.736	0.535<br>0.346-0.725	0.545<br>0.334-0.755	0.435<br>0.220-0.651	0.505<br>0.303-0.708	0.513<br>0.431-0.594	0.557<br>0.347-0.766	0.466<br>0.325-0.608	0.530<br>0.325-0.735	0.541<br>0.432-0.651	0.601<br>0.430-0.772	0.590<br>0.395-0.784	0.597<br>0.427-0.766	0.596<br>0.502-0.690	0.528<br>0.312-0.744
T3-test	0.522<br>0.322-0.722	0.476<br>0.298-0.655	0.623<br>0.427-0.819	0.534<br>0.331-0.737	0.712<br>0.556-0.868	0.573<br>0.496-0.651	0.643<br>0.453-0.834	0.588<br>0.403-0.774	0.598<br>0.370-0.825	0.610<br>0.503-0.716	0.713<br>0.574-0.853	0.774<br>0.666-0.881	0.726<br>0.608-0.844	0.738<br>0.673-0.803	0.711<br>0.555-0.866
T4-test	0.509<br>0.282-0.736	0.620<br>0.411-0.830	0.549<br>0.304-0.795	0.537<br>0.302-0.772	0.640<br>0.399-0.881	0.571<br>0.483-0.659	0.546<br>0.228-0.864	0.638<br>0.408-0.867	0.516<br>0.214-0.819	0.567<br>0.424-0.710	0.644<br>0.362-0.926	0.714<br>0.495-0.933	0.728<br>0.512-0.944	0.695<br>0.575-0.815	0.705<br>0.487-0.922
T5-test	0.646<br>0.494-0.798	0.639<br>0.504-0.775	0.733<br>0.621-0.844	0.643<br>0.478-0.809	0.775<br>0.688-0.863	0.687<br>0.632-0.743	0.660<br>0.495-0.825	0.789<br>0.696-0.882	0.638<br>0.440-0.836	0.696<br>0.610-0.781	0.742<br>0.603-0.881	0.782<br>0.697-0.868	0.831<br>0.785-0.877	0.785<br>0.732-0.838	0.809<br>0.738-0.880
int. avg.	0.511<br>0.438-0.584	0.496<br>0.425-0.567	0.554<br>0.475-0.632	0.478<br>0.397-0.558	0.585<br>0.508-0.662	0.525<br>0.491-0.558	0.534<br>0.450-0.618	0.585<br>0.511-0.659	0.517<br>0.429-0.605	0.546<br>0.499-0.592	0.620<br>0.548-0.693	0.640<br>0.569-0.710	0.653<br>0.585-0.720	0.638<br>0.598-0.678	0.621<br>0.546-0.696
T6-test	0.505<br>0.360-0.649	0.456<br>0.285-0.627	0.507<br>0.340-0.674	0.443<br>0.260-0.625	0.518<br>0.344-0.693	0.486<br>0.416-0.555	0.519<br>0.341-0.696	0.619<br>0.488-0.750	0.474<br>0.292-0.656	0.537<br>0.447-0.627	0.701<br>0.578-0.825	0.603<br>0.453-0.753	0.631<br>0.479-0.782	0.645<br>0.568-0.722	0.674<br>0.541-0.806
T7-test	0.556<br>0.421-0.692	0.530<br>0.382-0.677	0.608<br>0.413-0.803	0.557<br>0.380-0.734	0.612<br>0.441-0.782	0.573<br>0.505-0.641	0.595<br>0.403-0.786	0.640<br>0.493-0.788	0.584<br>0.392-0.776	0.606<br>0.511-0.702	0.682<br>0.536-0.829	0.623<br>0.470-0.775	0.687<br>0.527-0.847	0.664<br>0.582-0.746	0.649<br>0.486-0.811
T8-test	0.475<br>0.430-0.520	0.427<br>0.379-0.475	0.532<br>0.482-0.581	0.447<br>0.398-0.496	0.545<br>0.496-0.593	0.485<br>0.464-0.507	0.493<br>0.443-0.542	0.528<br>0.480-0.576	0.509<br>0.457-0.561	0.510<br>0.481-0.539	0.609<br>0.566-0.653	0.596<br>0.554-0.638	0.620<br>0.576-0.664	0.608<br>0.584-0.633	0.600<br>0.553-0.648
ext. avg.	0.484<br>0.443-0.524	0.437<br>0.393-0.481	0.535<br>0.490-0.581	0.455<br>0.410-0.500	0.548<br>0.503-0.592	0.492<br>0.472-0.511	0.502<br>0.456-0.548	0.544<br>0.501-0.587	0.512<br>0.464-0.560	0.519<br>0.493-0.546	0.622<br>0.583-0.661	0.599<br>0.560-0.637	0.626<br>0.585-0.666	0.616<br>0.593-0.638	0.610<br>0.567-0.653
avg.	0.490<br>0.455-0.526	0.452<br>0.415-0.489	0.540<br>0.501-0.579	0.461<br>0.422-0.500	0.557<br>0.519-0.595	0.500<br>0.483-0.517	0.510<br>0.470-0.551	0.554<br>0.517-0.591	0.513<br>0.471-0.555	0.526<br>0.503-0.549	0.622<br>0.587-0.656	0.609<br>0.575-0.643	0.633<br>0.598-0.667	0.621<br>0.601-0.641	0.613<br>0.576-0.650

Results are Dice values (higher is better) with 95% CI. int.: internal (T1-T5) test sets; ext.: external (T6-T8) test sets; ep.: episode; avg.: average.

Supplementary Table 5 Evaluation details on Brain Midline dataset.

	local						sequential				Relay			
	1	2	3	4	5	avg.	ep.1	ep.2	ep.3	avg.	ep.1	ep.2	ep.3	avg.
M1-test	1.192<br>1.034-1.349	5.228<br>4.219-6.237	2.805<br>2.343-3.268	4.470<br>3.508-5.432	3.327<br>2.868-3.786	3.404<br>3.092-3.717	3.439<br>2.952-3.925	2.101<br>1.828-2.375	2.574<br>2.107-3.041	2.705<br>2.461-2.948	2.117<br>1.905-2.330	2.024<br>1.622-2.427	1.814<br>1.463-2.166	1.985<br>1.794-2.177
M2-test	2.426<br>2.094-2.759	0.326<br>0.204-0.448	2.882<br>2.440-3.324	4.073<br>3.413-4.732	3.141<br>2.695-3.586	2.569<br>2.370-2.769	2.505<br>2.125-2.885	2.228<br>1.814-2.643	2.745<br>2.311-3.179	2.493<br>2.256-2.729	2.320<br>1.992-2.648	1.927<br>1.581-2.273	2.430<br>2.031-2.829	2.225<br>2.018-2.432
M3-test	2.443<br>1.730-3.156	3.843<br>2.852-4.834	0.428<br>0.264-0.592	1.752<br>1.167-2.336	2.033<br>1.606-2.460	2.100<br>1.811-2.389	1.829<br>1.494-2.165	3.058<br>2.106-4.011	0.546<br>0.284-0.809	1.811<br>1.459-2.163	1.772<br>1.443-2.102	2.225<br>1.624-2.825	0.791<br>0.207-1.376	1.596<br>1.295-1.897
M4-test	1.968<br>1.325-2.611	3.024<br>1.415-4.632	2.472<br>1.498-3.445	0.050<br>0.000-0.101	1.466<br>0.963-1.969	1.796<br>1.384-2.208	1.984<br>1.282-2.685	1.619<br>1.108-2.129	3.414<br>1.871-4.958	2.339<br>1.749-2.929	1.742<br>1.086-2.399	2.081<br>1.163-3.000	1.698<br>1.190-2.207	1.841<br>1.430-2.251
M5-test	4.793<br>3.892-5.693	9.375<br>7.608-11.140	3.076<br>2.480-3.671	4.152<br>3.287-5.016	0.993<br>0.501-1.485	4.477<br>4.006-4.949	0.905<br>0.435-1.375	4.508<br>3.422-5.593	3.114<br>2.371-3.856	2.842<br>2.371-3.313	0.724<br>0.536-0.911	2.632<br>1.962-3.302	2.065<br>1.599-2.531	1.807<br>1.525-2.089
int. avg.	2.524<br>2.279-2.770	4.205<br>3.702-4.709	2.487<br>2.254-2.721	3.519<br>3.136-3.902	2.464<br>2.241-2.687	3.041<br>2.890-3.192	2.298<br>2.084-2.512	2.725<br>2.423-3.027	2.484<br>2.217-2.751	2.502<br>2.350-2.654	1.819<br>1.678-1.960	2.151<br>1.919-2.382	1.879<br>1.675-2.084	1.950<br>1.837-2.063
M6-test	4.224<br>3.843-4.604	10.870<br>9.739-12.000	4.757<br>4.324-5.190	5.241<br>4.746-5.735	4.524<br>4.173-4.875	5.923<br>5.633-6.214	4.795<br>4.417-5.174	4.150<br>3.661-4.639	5.199<br>4.649-5.749	4.715<br>4.439-4.991	3.320<br>2.992-3.648	3.478<br>3.061-3.895	3.703<br>3.319-4.087	3.500<br>3.282-3.718
M7-test	2.426<br>1.679-3.173	2.975<br>2.188-3.761	2.096<br>1.329-2.864	2.426<br>1.599-3.254	2.130<br>1.293-2.968	2.411<br>2.062-2.760	2.313<br>1.542-3.084	2.133<br>1.337-2.929	1.910<br>1.152-2.669	2.119<br>1.679-2.559	2.112<br>1.444-2.780	2.232<br>1.692-2.772	2.347<br>1.608-3.085	2.230<br>1.859-2.601
M8-test	2.213<br>1.779-2.647	3.761<br>3.154-4.369	2.962<br>2.423-3.502	3.002<br>2.508-3.495	3.556<br>2.898-4.214	3.099<br>2.851-3.346	3.856<br>3.177-4.535	2.160<br>1.751-2.570	2.831<br>2.263-3.400	2.949<br>2.621-3.278	2.202<br>1.932-2.471	1.903<br>1.716-2.089	2.007<br>1.824-2.190	2.037<br>1.912-2.162
ext. avg.	3.830<br>3.514-4.146	9.394<br>8.469-10.320	4.347<br>3.986-4.707	4.757<br>4.349-5.165	4.249<br>3.947-4.550	5.315<br>5.078-5.553	4.519<br>4.197-4.842	3.747<br>3.348-4.147	4.670<br>4.217-5.123	4.312<br>4.084-4.541	3.090<br>2.821-3.358	3.179<br>2.842-3.516	3.380<br>3.067-3.692	3.216<br>3.039-3.393
avg.	2.723<br>2.543-2.902	5.913<br>5.451-6.374	3.099<br>2.901-3.298	3.926<br>3.636-4.217	3.052<br>2.870-3.233	3.742<br>3.614-3.870	3.029<br>2.847-3.210	3.062<br>2.820-3.303	3.203<br>2.967-3.438	3.098<br>2.970-3.225	2.237<br>2.106-2.368	2.489<br>2.298-2.680	2.375<br>2.202-2.547	2.367<br>2.271-2.463

Results are HD values (lower is better) with 95% CI. int.: internal (M1-M5) test sets; ext.: external (M6-M8) test sets; ep.: episode; avg.: average.

Supplementary Table 6 Evaluation details compared to Federated Learning and Swarm Learning.

	Federated	Swarm	central	Relay		Federated	Swarm	central	Relay
F1-test	0.783<br>0.747-0.818	0.789<br>0.748-0.829	0.836<br>0.808-0.863	0.787<br>0.763-0.812	M1-test	0.433<br>0.275-0.591	0.475<br>0.323-0.628	0.476<br>0.320-0.632	0.492<br>0.339-0.645
F2-test	0.714<br>0.662-0.767	0.683<br>0.620-0.746	0.786<br>0.750-0.821	0.751<br>0.715-0.787	M2-test	0.522<br>0.302-0.741	0.523<br>0.306-0.740	0.528<br>0.312-0.744	0.601<br>0.430-0.772
F3-test	0.856<br>0.839-0.873	0.877<br>0.861-0.892	0.887<br>0.871-0.903	0.873<br>0.858-0.888	M3-test	0.724<br>0.578-0.870	0.701<br>0.534-0.867	0.711<br>0.555-0.866	0.713<br>0.574-0.853
F4-test	0.841<br>0.826-0.857	0.855<br>0.839-0.870	0.892<br>0.878-0.905	0.880<br>0.867-0.894	M4-test	0.705<br>0.491-0.918	0.680<br>0.456-0.905	0.705<br>0.487-0.922	0.644<br>0.362-0.926
					M5-test	0.779<br>0.685-0.872	0.803<br>0.717-0.889	0.809<br>0.738-0.880	0.742<br>0.603-0.881
int. avg.	0.807<br>0.790-0.823	0.811<br>0.560-0.592	0.856<br>0.844-0.868	0.832<br>0.819-0.845	int. avg.	0.603<br>0.526-0.681	0.614<br>0.538-0.690	0.621<br>0.546-0.696	0.620<br>0.548-0.693
F5-test	0.616<br>0.600-0.631	0.576<br>0.792-0.830	0.583<br>0.566-0.600	0.747<br>0.736-0.758	M6-test	0.608<br>0.440-0.775	0.668<br>0.540-0.795	0.674<br>0.541-0.806	0.701<br>0.578-0.825
					M7-test	0.604<br>0.425-0.784	0.618<br>0.444-0.793	0.649<br>0.486-0.811	0.682<br>0.536-0.829
					M8-test	0.563<br>0.514-0.613	0.590<br>0.542-0.638	0.600<br>0.553-0.648	0.609<br>0.566-0.653
					ext. avg.	0.570<br>0.525-0.615	0.598<br>0.555-0.641	0.610<br>0.567-0.653	0.622<br>0.583-0.661
avg.	0.672<br>0.659-0.685	0.645<br>0.631-0.660	0.663<br>0.649-0.678	0.772<br>0.763-0.781	avg.	0.578<br>0.540-0.617	0.602<br>0.565-0.639	0.613<br>0.576-0.650	0.622<br>0.587-0.656

Dice (higher is better) results on Retina Fundus (F1-F5) and Mediastinum Tumor (M1-M8) datasets are shown with 95% CI. The result of Relay Learning is on episode 1 here. int.: internal test sets; ext.: external test sets; avg.: average.

Supplementary Table 7 Statistical analysis detail.

int. F-test	local 1	local 2	local 3	local 4	
Relay vs.	< 0.001 (***)	< 0.001 (***)	< 0.001 (***)	< 0.001 (***)	

Relay vs. sequential		Relay		Relay vs.	
ep. 1	< 0.001 (***)	ep. 1 vs. ep. 2	0.952 (ns)	Federated	0.055 (ns)
ep. 2	< 0.001 (***)	ep. 1 vs. ep. 2	> 0.999 (ns)	Swarm	0.134 (ns)
ep. 3	< 0.001 (***)	ep.2 vs. ep. 3	0.946 (ns)	central	0.071 (ns)

ext. F-test	local 1	local 2	local 3	local 4	
Relay vs.	< 0.001 (***)	< 0.001 (***)	< 0.001 (***)	< 0.001 (***)	

Relay vs. sequential		Relay		Relay vs.	
ep. 1	< 0.001 (***)	ep. 1 vs. ep. 2	0.130 (ns)	Federated	< 0.001 (***)
ep. 2	< 0.001 (***)	ep. 1 vs. ep. 3	0.002 (**)	Swarm	< 0.001 (***)
ep. 3	< 0.001 (***)	ep. 2 vs. ep. 3	< 0.001 (***)	central	< 0.001 (***)

int. T-test	local 1	local 2	local 3	local 4	local 5
Relay vs.	0.120 (ns)	0.081 (ns)	0.382 (ns)	0.040 (*)	0.512 (ns)

Relay vs. sequential		Relay		Relay vs.	
ep. 1	0.304 (ns)	ep. 1 vs. ep. 2	0.931 (ns)	Federated	0.978 (ns)
ep. 2	0.672 (ns)	ep. 1 vs. ep. 3	0.823 (ns)	Swarm	0.999 (ns)
ep. 3	0.038 (*)	ep. 2 vs. ep. 3	0.970 (ns)	central	> 0.999 (ns)

ext. T-test	local 1	local 2	local 3	local 4	local 5
Relay vs.	< 0.001 (***)	< 0.001 (***)	0.016 (*)	< 0.001 (***)	0.032 (*)

Relay vs. sequential		Relay		Relay vs.	
ep. 1	< 0.001 (***)	ep. 1 vs. ep. 2	0.730 (ns)	Federated	0.211 (ns)
ep. 2	0.204 (ns)	ep. 1 vs. ep. 3	0.992 (ns)	Swarm	0.776 (ns)
ep. 3	< 0.001 (***)	ep. 2 vs. ep. 3	0.654 (ns)	central	0.959 (ns)

int. M-test	local 1	local 2	local 3	local 4	local 5
Relay vs.	0.008 (**)	< 0.001 (***)	0.013 (*)	< 0.001 (***)	0.018 (*)

Relay vs. sequential		Relay	
ep. 1	0.013 (*)	ep. 1 vs. ep. 2	0.117 (ns)
ep. 2	0.002 (**)	ep. 1 vs. ep. 3	0.931 (ns)
ep. 3	< 0.001 (***)	ep. 2 vs. ep. 3	0.237 (ns)

ext. M-test	local 1	local 2	local 3	local 4	local 5
Relay vs.	0.131 (ns)	< 0.001 (***)	0.002 (**)	< 0.001 (***)	0.004 (**)

Relay vs. sequential		Relay	
ep. 1	< 0.001 (***)	ep. 1 vs. ep. 2	0.979 (ns)
ep. 2	0.076 (ns)	ep. 1 vs. ep. 3	0.587 (ns)
ep. 3	< 0.001 (***)	ep. 2 vs. ep. 3	0.817 (ns)

The statistics are P-values in Retina Fundus (F-test), Mediastinum Tumor (T-test), and Brain Midline (M-test) datasets. int.: internal test sets; ext.: external test sets; ep.: episode; vs. versus; ns: $P \geq 0.05$; *: $P < 0.05$; **: $P < 0.01$; ***: $P < 0.001$.

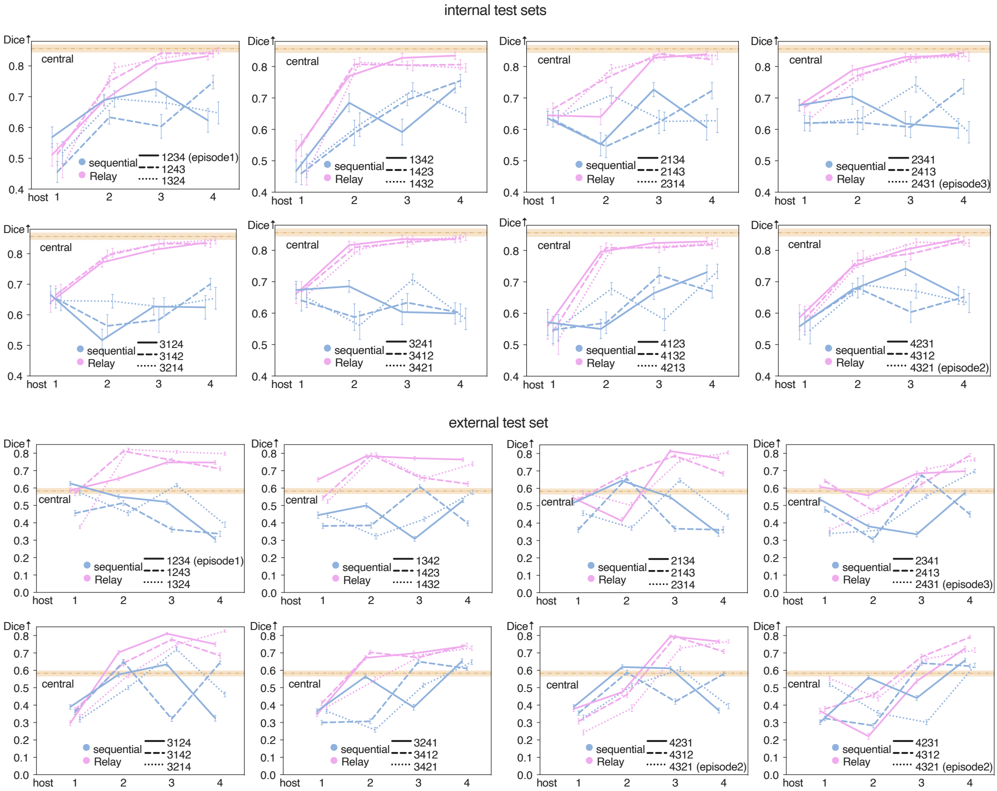

Supplementary Fig. 1 Chronology analysis of all training order permutations on Retina Fundus dataset. The horizontal axis shows the training sequence, while the training order of different institutions is shown in the legend. The Dice value with 95% CI was computed on the internal and external test sets of the Retina Fundus dataset. ↑: higher is better.

Supplementary Table 8 Final Dice scores of all training order permutations on the internal test sets of the Retina Fundus dataset.

order	1234	1243	1324	1342	1423	1432
sequential	0.623<br>0.585-0.662	0.748<br>0.726-0.770	0.648<br>0.612-0.683	0.732<br>0.717-0.747	0.755<br>0.735-0.775	0.646<br>0.622-0.670
Relay	0.832<br>0.819-0.845	0.840<br>0.829-0.852	0.850<br>0.840-0.860	0.835<br>0.823-0.847	0.806<br>0.790-0.822	0.796<br>0.782-0.809
order	2134	2143	2314	2341	2413	2431
sequential	0.606<br>0.566-0.647	0.723<br>0.697-0.749	0.628<br>0.590-0.665	0.603<br>0.572-0.635	0.736<br>0.713-0.759	0.591<br>0.557-0.625
Relay	0.839<br>0.829-0.849	0.822<br>0.807-0.837	0.835<br>0.822-0.848	0.836<br>0.823-0.848	0.844<br>0.833-0.854	0.832<br>0.818-0.847
order	3124	3142	3214	3241	3412	3421
sequential	0.625<br>0.586-0.663	0.701<br>0.682-0.720	0.655<br>0.619-0.690	0.599<br>0.566-0.633	0.601<br>0.573-0.630	0.582<br>0.548-0.616
Relay	0.836<br>0.825-0.847	0.834<br>0.820-0.847	0.844<br>0.833-0.855	0.835<br>0.824-0.846	0.838<br>0.826-0.849	0.843<br>0.832-0.854
order	4123	4132	4213	4231	4312	4321
sequential	0.731<br>0.708-0.754	0.669<br>0.649-0.689	0.734<br>0.710-0.757	0.656<br>0.628-0.684	0.651<br>0.627-0.676	0.632<br>0.602-0.663
Relay	0.829<br>0.816-0.841	0.819<br>0.806-0.832	0.824<br>0.812-0.836	0.836<br>0.824-0.848	0.830<br>0.816-0.843	0.823<br>0.812-0.834

Results are shown with 95% CI. order: the host order in the training of Relay Learning, e.g., "1234" means "F1-F2-F3-F4".

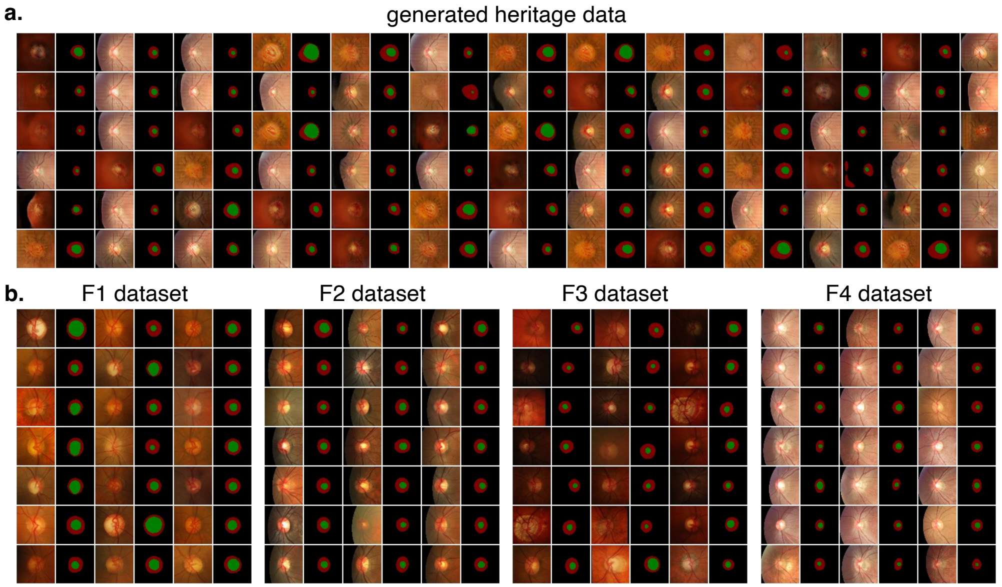

Supplementary Fig. 2 Visualization of the DoubleGAN-based relay system on the Retina Fundus dataset. **a**, Generated fake heritage samples by DoubleGAN. **b**, Real samples from each internal site of the dataset. The samples are randomly selected, and both the images and the segmentation label maps are shown. Colors in label maps: green (optic cup), red (optic disk), and black (background).

Supplementary Table 9 Comparison to compatible methods and analysis on two-stage strategy.

	sequential	SI	OWM	Relay (one-stage)	Relay (two-stage)
F1-test	0.349<br>0.298-0.400	0.354<br>0.293-0.415	0.320<br>0.261-0.380	0.699<br>0.663-0.736	0.787<br>0.763-0.812
F2-test	0.250<br>0.177-0.323	0.273<br>0.193-0.352	0.269<br>0.198-0.339	0.572<br>0.512-0.633	0.751<br>0.715-0.787
F3-test	0.818<br>0.794-0.841	0.829<br>0.808-0.849	0.808<br>0.784-0.833	0.838<br>0.821-0.855	0.873<br>0.858-0.888
F4-test	0.884<br>0.868-0.899	0.885<br>0.872-0.899	0.886<br>0.872-0.899	0.882<br>0.866-0.898	0.880<br>0.867-0.894
int. avg.	0.623<br>0.585-0.662	0.633<br>0.594-0.672	0.620<br>0.581-0.659	0.766<br>0.744-0.788	0.832<br>0.819-0.845
F5-test/<br>ext. avg.	0.306<br>0.290-0.322	0.285<br>0.269-0.301	0.219<br>0.204-0.234	0.554<br>0.540-0.568	0.747<br>0.736-0.758
avg.	0.399<br>0.381-0.418	0.388<br>0.369-0.407	0.337<br>0.318-0.357	0.616<br>0.603-0.630	0.772<br>0.763-0.781

Results are mean Dice values (higher is better) with 95% CI of the main episode training on the Retina Fundus dataset (F1-F4). avg.: average.

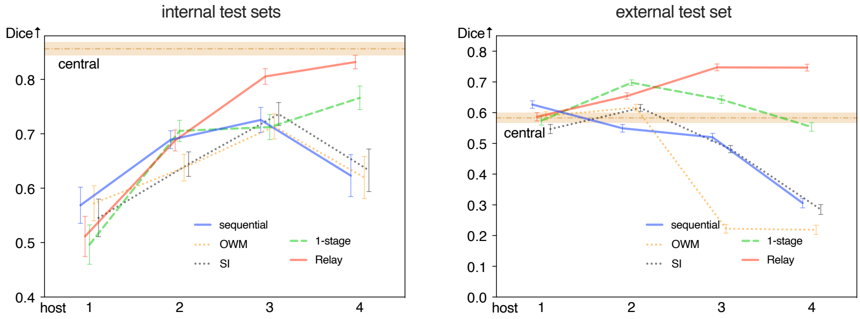

Supplementary Fig. 3 Comparison to compatible methods (SI and OWM) and analysis of two-stage strategy. By default, Relay Learning used the two-stage strategy. The horizontal axis shows the training sequence, and different methods are shown in the legend. The mean Dice value with 95% CI was computed using the main episode. ↑: higher is better.
